# Ancestral Chaperonins Provide the First Structural Glimpse into Early Multimeric Protein Evolution

**DOI:** 10.1093/molbev/msaf314

**Published:** 2025-11-28

**Authors:** Rita Severino, Jorge Cuéllar, Jorge Gutiérrez-Seijo, Moisés Maestro-López, Luis Sánchez-Pulido, César Santiago, Mercedes Moreno-Paz, José María Valpuesta, Víctor Parro

**Affiliations:** Centro de Astrobiología (CAB), INTA-CSIC, Madrid, Spain; Space Research and Astrobiology PhD Program, University of Alcalá (UAH), Madrid, Spain; National Center for Biotechnology (CNB), CSIC, Madrid, Spain; National Center for Biotechnology (CNB), CSIC, Madrid, Spain; National Center for Biotechnology (CNB), CSIC, Madrid, Spain; Centro de Astrobiología (CAB), INTA-CSIC, Madrid, Spain; National Center for Biotechnology (CNB), CSIC, Madrid, Spain; Centro de Astrobiología (CAB), INTA-CSIC, Madrid, Spain; National Center for Biotechnology (CNB), CSIC, Madrid, Spain; Unidad de Nanobiotecnología, CNB-CSIC-IMDEA Nanociencia Associated Unit, Madrid, Spain; Centro de Astrobiología (CAB), INTA-CSIC, Madrid, Spain

**Keywords:** Protein resurrection, chaperonins, ASR, cryoEM, oligomeric states, ATPase activity, evolutionary intermediates, multimeric complexity

## Abstract

Chaperonins are essential protein-folding machines, classified into three structural and phylogenetic groups: Group I (bacterial GroEL), Group II (archaeal thermosome and eukaryotic CCT), and Group III (bacterial thermosome-like). Using ancestral sequence reconstruction (ASR) and protein resurrection, we inferred and experimentally characterized the last common ancestors of these groups (ancestral chaperonins ACI, ACII, and ACIII). The resurrected proteins exhibited ATPase activity (except ACII) and protected client proteins from heat-induced inactivation. Structural analyses by electron microscopy and Cryo-EM revealed that ACI forms single 7-mer rings, whereas ACII adopts a mixed population of single/double 8-mer rings, representing the first experimental observation of intermediate oligomeric states. ACII also features a unique cochaperonin-independent closure mechanism, distinct from modern Group I and II chaperonins. Together, these findings provide the experimental structural reconstruction of the most ancient and complex multimeric proteins so far, uncover novel intermediate states in chaperonin evolution, and offer a direct empirical framework for studying the emergence of multimeric complexity in early cellular life.

## Introduction

Protein folding is a cornerstone of cellular function and overall organismal health, as misfolded or aggregated proteins can cause various cellular malfunctions. At the core of this process are a large group of proteins known as molecular chaperones. Among them, chaperonins (also known as Hsp60 chaperones) are a ubiquitous family across all life kingdoms. These large, barrel-shaped complexes act as molecular machines, providing a protected environment for newly synthesized proteins to fold efficiently, a critical aspect of protein homeostasis (Horwich and Fenton 2020; Stan et al. 2022; Horovitz et al. 2024). Beyond folding, chaperonins also defend against stress (eg temperature fluctuations, oxidative stress, or chemical exposure) by preventing aggregation and assisting in refolding misfolded proteins (Llorca et al. 1998; Rowland and Robb 2017; Santra et al. 2018; Storey and Storey 2023).

Chaperonins likely evolved from a multifunctional ancestor within the thioredoxin superfamily, specifically a peroxiredoxin-like protein with both antioxidant and chaperone activities (Dekker et al. 2011; Rowland and Robb 2017). This dual functionality persists in modern thioredoxin-domain containing *chaperedoxins* (which associate with GroEL in vivo) and peroxiredoxins, highlighting their role in shielding proteins and DNA from oxidative and thermal stress (Goemans et al. 2018; Stroobants et al. 2019; Muñoz-Villagrán et al. 2024). This evolutionary origin suggests that extreme environmental pressures (eg high temperature, acidity, variable salinity) shaped early chaperonins. The widespread presence and persistence of chaperonins and thioredoxins in bacterial genomes, often under positive selection, underscores their evolutionary importance and adaptability (Bresell and Persson 2007).

All chaperonin monomers share a common three-domain structure (Fig. S1): an apical domain involved in substrate recognition, an equatorial domain containing the ATP-binding site that drives the conformational changes that occur during the chaperonin functional cycle, and an intermediate domain linking the two by relaying signals. Despite this conserved framework (Fig. S1a), chaperonins diverge into three structurally distinct groups (Groups I–III) based on their unique architecture and phylogeny (Rowland and Robb 2017; Horwich and Fenton 2020; Stan et al. 2022; Horovitz et al. 2024). This structural diversity raises intriguing questions about their evolutionary origins, including how their common ancestor emerged and how they diverged.

Group I chaperonins, found in bacteria and eukaryotic organelles (eg mitochondria and chloroplasts), are exemplified by the well-studied GroEL/GroES system of *Escherichia coli*. GroEL consists of two stacked rings, each comprising seven identical subunits (∼60 kDa), while GroES (∼10 kDa) forms a homoheptamer that caps the GroEL folding chamber, acting as a cochaperonin. Group II chaperonins, present in archaea and the eukaryotic cytosol (eg the thermosome in archaea and the CCT/TRiC complex in eukaryotes), feature a built-in “lid” mechanism, eliminating the need for a separate cochaperonin. Group II chaperonins have evolved from rings composed of one to three different subunits in the archaeal thermosome, to eight distinct subunits in the eukaryotic CCT (CCT1-CCT8) (Horovitz et al. 2024). Group III chaperonins, found in some bacteria, resemble Group II but lack the nucleotide-sensing loop present in Groups I and II (Techtmann and Robb 2010; An et al. 2017) (Figure S1b). This conserved amino acid sequence directly influences the rate of nucleotide hydrolysis, thereby controlling the timing of the folding cycle. The nucleotide-sensing loop undergoes conformational changes that regulate the opening and closing of the chaperonin rings and facilitates communication by transmitting ATP hydrolysis signals between subunits (Pereira et al. 2012). The structural differences between these groups suggest different evolutionary paths, yet they are related by homology and function (Dekker et al. 2011).

The evolutionary relationships between these groups remain unresolved. Some researchers propose that Group III chaperonins represent an ancient form from which Groups I and II originated, with GroES possibly arising from the excision of the lid region of a Group III chaperonin (An et al. 2017). Others argue that Group III chaperonins emerged later, possibly through horizontal gene transfer from an ancestral archaeon to a bacterial Firmicutes (Techtmann and Robb 2010; Rebeaud et al. 2021). These competing hypotheses highlight the complexity of chaperonin evolution and the need for new approaches to unravel their history.

Ancestral sequence reconstruction (ASR), first proposed in 1963 by Pauling and Zuckerkandl (Pauling and Zuckerkandl 1963), is a powerful tool to address these questions. By inferring the sequences of ancient chaperonins and then “resurrecting” them in the laboratory, we can gain insights into the structural and functional properties of these molecular machines in early life (Hochberg and Thornton 2017; Kaçar 2024). ASR followed by protein production, also known as “protein resurrection,” has been successfully applied to other ancient proteins, revealing trends such as increasing promiscuity, greater conformational diversity, higher thermal stability, and improved heterologous expression (Wheeler et al. 2016; Gamiz-Arco et al. 2021). For example, ASR studies of ancient thioredoxins, nitrogenases, β-lactamases, V-ATPases, elongation factor Tu, and RuBisCO, have provided valuable insights into the evolution of protein complexity and function (Gaucher et al. 2003; Finnigan et al. 2012; Ingles-Prieto et al. 2013; Risso et al. 2015; Shih et al. 2016; Schulz et al. 2022; Harris et al. 2024; Amritkar et al. 2025).

In this study, we apply ASR to chaperonins to explore their evolutionary origins and structural diversification. By rebuilding ancestral chaperonin (AC) sequences and examining their oligomeric structures, we aim to answer key questions: which group of chaperonins evolved first, and how did the structural differences between Groups I, II, and III emerge over evolutionary time? Beyond shedding light on chaperonin evolution, these findings are expected to offer insights into how early life may have adapted to extreme environments on Earth and potentially on other planets (Severino et al. 2023).

## Results

### Reconstruction and Characterization of Ancestral Chaperonins

**Fig. 1.**
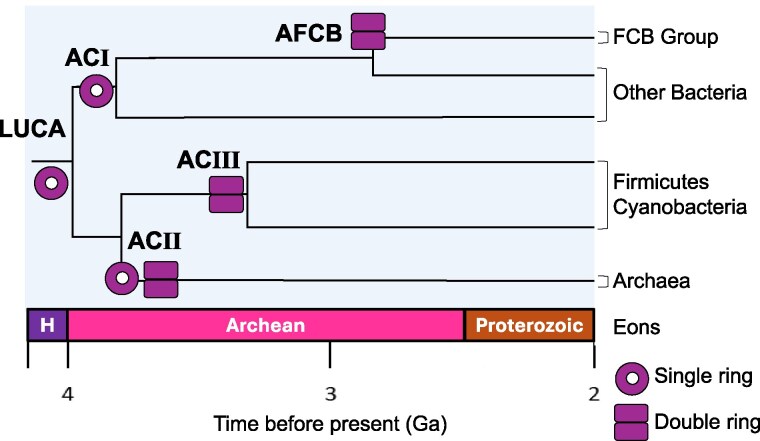
Evolutionary history of prokaryotic chaperonins. Hypothetical evolutionary trajectory of ancestral chaperonins from single- to double-ring structures. Nodes ACI (single-ring), ACII (single- and double-ring), ACIII (double-ring) and AFCB (double-ring) are shown. Divergence times are approximate and based on data from the TimeTree project (Kumar et al. 2022). H—Hadean.

To better understand the evolutionary history of chaperonins—one of the oldest protein families found across of all life forms—we reconstructed their phylogenetic history using 96 representative prokaryotic chaperonin sequences (Fig. S2). In the absence of an outgroup, the precise location of the last unknown common ancestor (LUCA) cannot be identified, although the classical view, as well as recent research, suggest it lies somewhere on the branch connecting bacterial and archaeal populations (eg Moody et al. 2024). We selected several AC nodes for protein synthesis and purification, including the last common ancestors of Group I (ACI), Group II (ACII), and Group III (ACIII), along with the ancestor of the Fibrobacteres–Chlorobi–Bacteroidetes (FCB) Group (AFCB) (Fig. 1, Figs. S2 and S3). We selected the FCB group as a more modern ancestor reference because of its well-definition in the phylogenetic tree, and because of its peculiarity of encoding a putative archaeal pathway for ether-bound isoprenoid membrane lipids besides the bacterial fatty acid membrane pathway, that support the hypothesis that LUCA possessed a heterochiral “mixed archaeal/bacterial membrane”, possibly a remnant from before bacteria and archaea diverged (Villanueva et al. 2021). The predicted ancestral chaperonins (AC) sequences showed a sequence identity of 60% to 81% when compared with modern homologs (see multiple sequence alignment of best NCBI BLAST matches in Figure S4) and were dated using data from the TimeTree project (Kumar et al. 2022) (Table 1).

**Table 1.** Best NCBI BLAST matches for resurrected ancestral chaperonin sequences

		Best hits (nr-NCBI database)	
	TimeTree Estimated age (Ga)	Identity	Taxon
**ACI**—Last Common Ancestor of Group I, GroEL	3.6–4.2	60%	*Alicyclobacillus sendaiensis* (WP_062305661.1)
**ACII**—Last Common Ancestor of Group II, thermosome	3.8–4.1	64%	*Thermococcus alcaliphilus* (WP_252742870.1)
**ACIII**—Last Common Ancestor of Group III, thermosome-like	∼3.0	67%	*Thermosinus carboxydivorans* (WP_007289467.1)
**AFCB**—Last Common Ancestor of FCB Group, GroEL	2.6–3.1	81%	*Rhodothermaceae bacterium* (GIV58090.1)

**Fig. 2.**
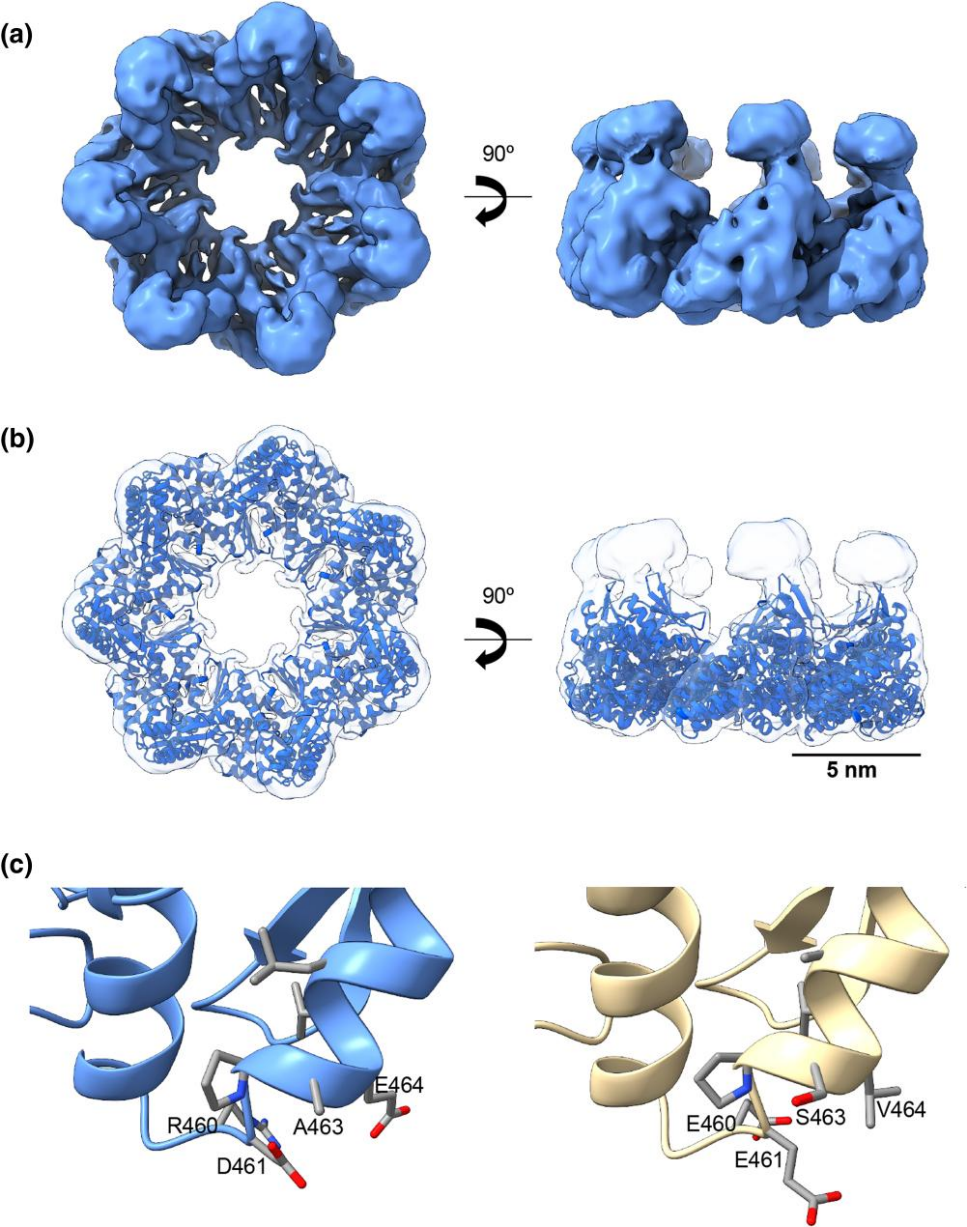
Cryo-EM structure of ACI. **a**) Top and side view of the 3D reconstruction of ACI (cornflower blue) at 5.4 Å resolution (EMD-54342). **b**) Docking of the ACI atomic model, generated by AlphaFold3, into the ACI 3D reconstruction. **c**) Conservation of residues R460, D461, A463, and E464 in ACI (blue) compared with the *E. coli* single-ring mutant (SR1), where these residues are present. By contrast, these residues are absent in *E. coli* GroEL (yellow). The presence of these residues in ACI prevents the formation of the inter-subunit interface, and thus disrupts the double-ring formation.

**Fig. 3.**
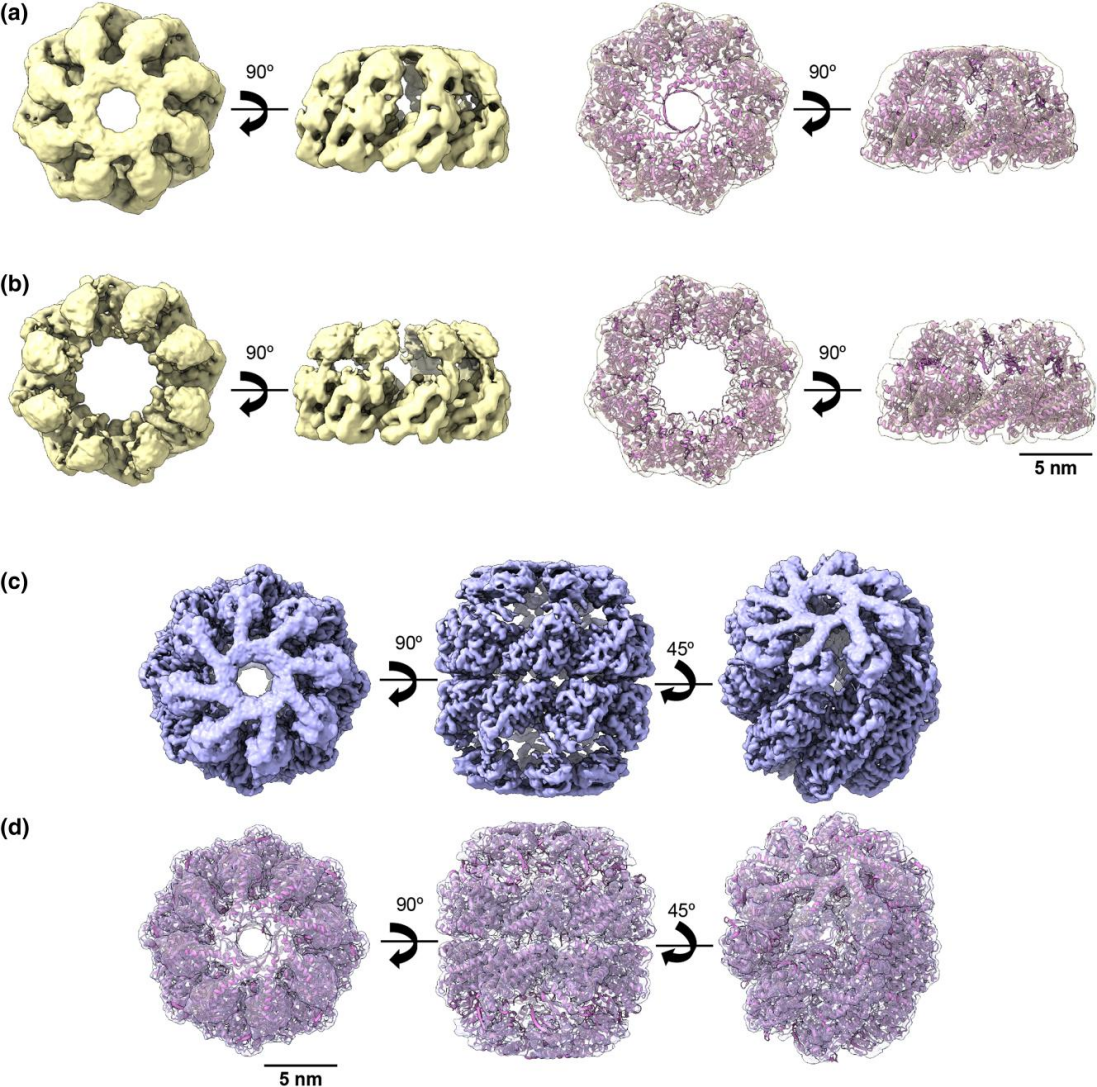
Cryo-EM structure of the ACII double and single rings. **a, b**) Left: Top and side view of the 3D reconstruction of ACII in a closed (a) (EMD-54344) and open (b) (EMD-54345) single-ring conformation (yellow). Right: Docking of the ACII atomic model, generated by AlphaFold3, into the 3D reconstruction of the open (a) and closed (b) ACII single ring. **c**) Top, side, and tilted view of the 3D reconstruction of ACII in a double-ring shape (purple) at 3.6 Å resolution (EMD-54339). **d**) Docking of the ACII atomic model, generated by AlphaFold3, into the 3D-reconstruction of the ACII double-ring (PDB: 9RWP).

**Fig. 4.**
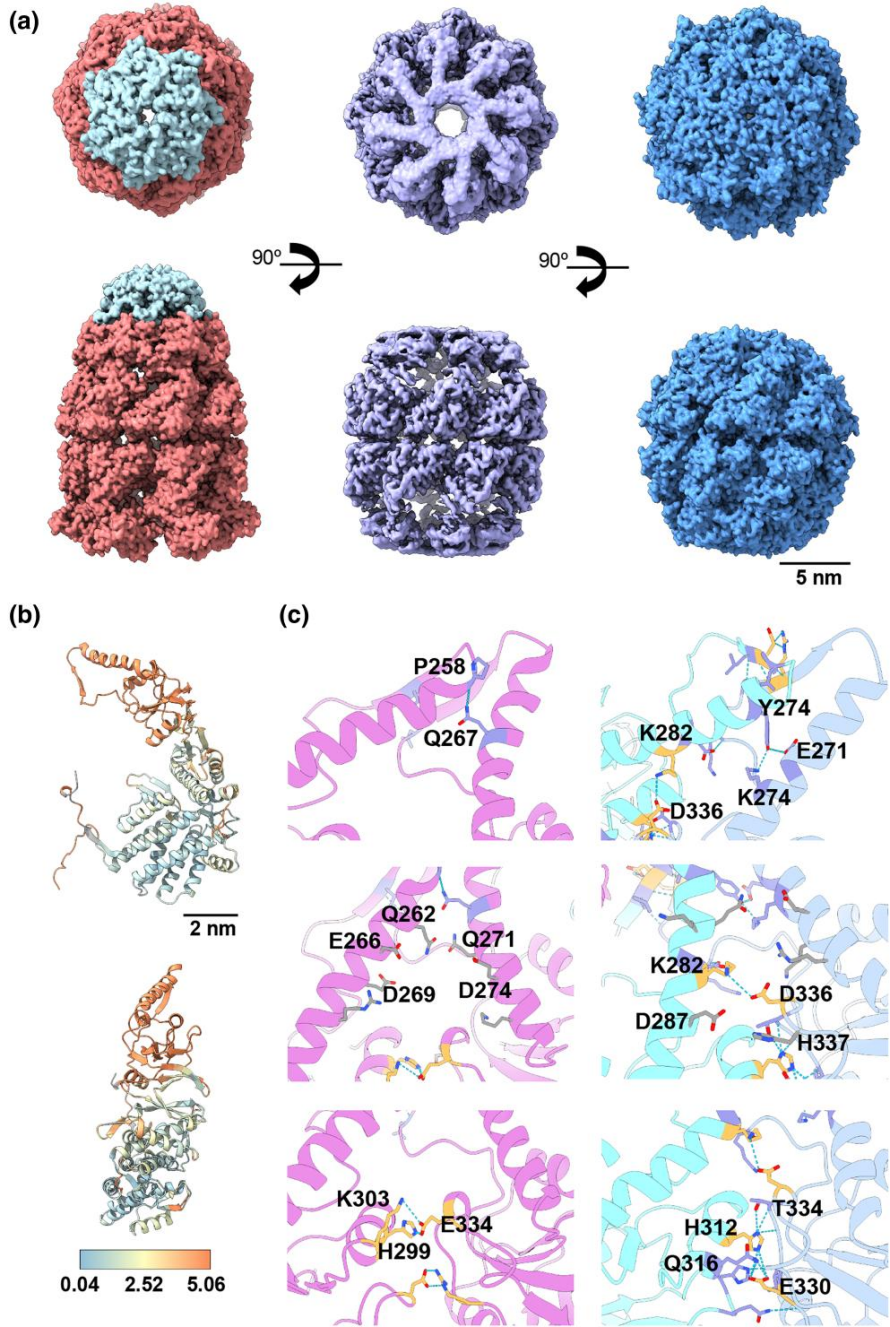
Structural comparison of ACII with GroEL and CCT. **a**) 3D reconstructions in two orthogonal orientations of *left* GroEL-GroES complex (Group I chaperonin); *center* ACII; and *right* CCT (group II chaperonin). The upper row shows top views, while the lower row displays the side views. The complexes are color-coded: red and cyan for *E. coli* GroEL (PDB: 8WUX), purple for ACII (PDB: 9RWP), and blue for CCT (PDB ID: 8SHG). **b**) Structural analysis of ACII and CCT. The two views depict the root-mean-square deviation (RMSD) comparison between the ACII subunit and CCT5 of the CCT complex, with a color gradient indicating structural differences. **c**) Three different views highlighting differences in the atomic interactions within the apical regions of ACII (pink) and CCT (cyan for CCT5 and steel blue for CCT2). The top panel shows the tip of the helical protrusion, the middle panel displays the base of the helical protrusion, and the bottom panel focuses on the apical domain helix. Orange residues represent salt bridges, while purple residues indicate hydrogen bonds.

**Fig. 5.**
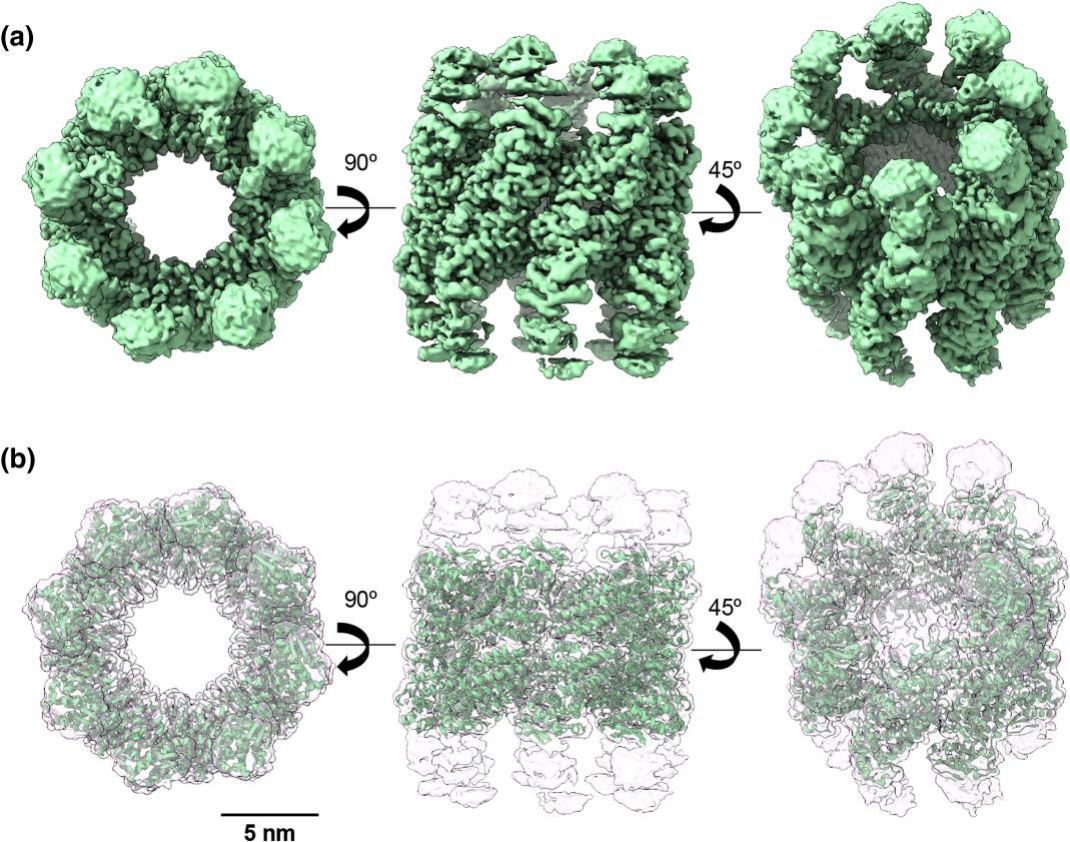
Cryo-EM structure of ACIII. **a**) Top, side, and tilted view of the 3D reconstruction of ACIII (light green) at 3.2 Å resolution (EMD-54340). **b**) Docking of the ACIII atomic model, generated by AlphaFold3, into the ACIII 3D reconstruction (PDB: 9RWQ). Note that only the equatorial domains were modeled.

**Fig. 6.**
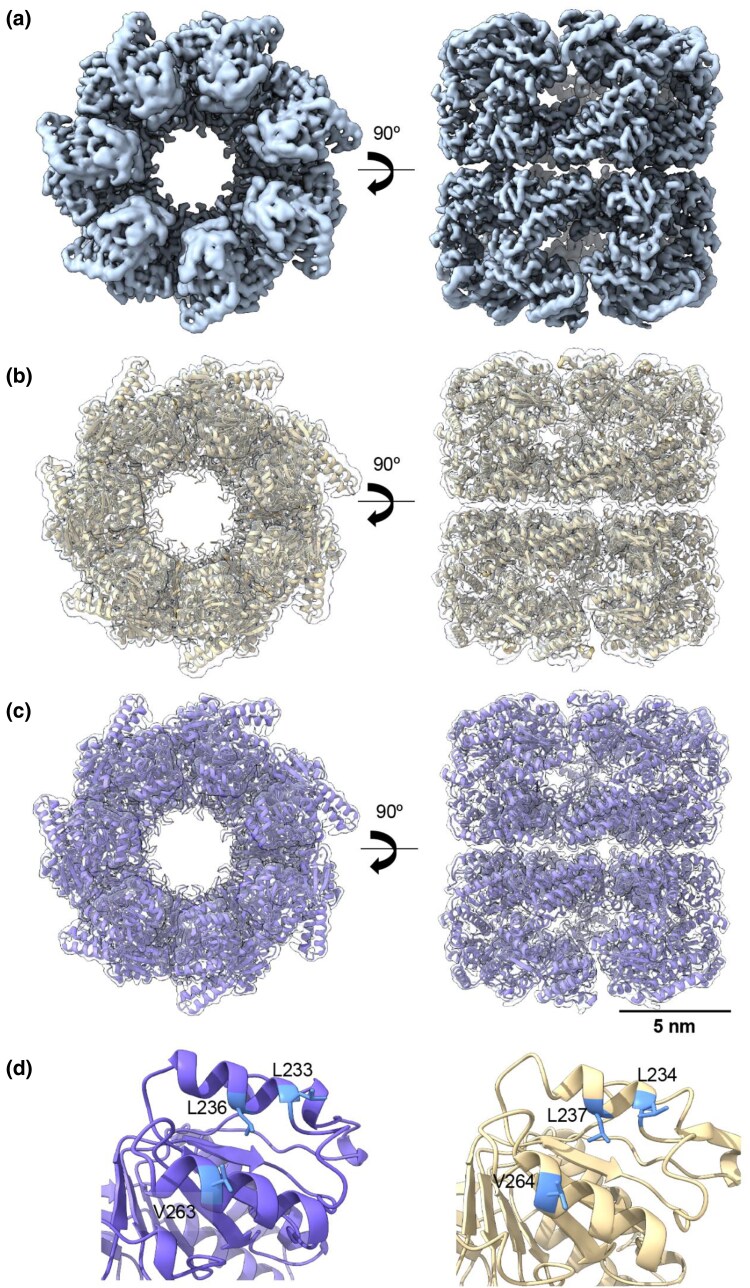
Cryo-EM structure of AFCB. **a**) Top and side view of the 3D reconstruction of AFCB (light steel blue) at 3.5 Å resolution (EMD-54341). **b**) Docking of the *E. coli* GroEL atomic model (PDB: 8WUX) into the AFCB 3D-reconstruction. **c**) Docking of the AFCB atomic model, generated by AlphaFold3, into the AFCB 3D-reconstruction (PDB: 9RWR). **d**) Conservation of L233, L236 and V263 as key residues for interaction with the cochaperonin GroES in AFCB (purple) and *E. coli* GroEL (yellow).

*E. coli* BL21 (DE3) cells were transformed with plasmids encoding four ancestral sequences, and the recombinant proteins were expressed and purified as described in Materials and Methods (Fig. S5). We first studied how these purified AC proteins assembled using transmission electron microscopy. ACI formed a seven-subunit single-ring oligomer but only in the presence of ATP-Mg^2+^. ACII formed a heterogeneous mixture of eight-subunit oligomers, including both single and double rings, even without ATP. Addition of ATP-Mg^2+^ promoted oligomerization of ACII, increasing overall ring formation. However, under the conditions we tested, it did not significantly alter the ratio between single and double rings. Single rings were consistently observed with a lower abundance, and their visualization and processing were only efficient when we increased the sample concentration. This suggests that while ATP-Mg^2+^ enhances oligomer formation, it does not preferentially stabilize one ring form over the other. ACIII assembled as eight-subunit double rings without ATP-Mg²^+^, and AFCB formed 7-subunit double rings (Figs. 2–6). Oligomers, as described in the text, did not require the presence of ATP to remain stable.

### Structural Characterization of Ancestral Chaperonins by Cryoelectron Microscopy

To investigate the structural details of the new proteins, we used cryoelectron microscopy (CryoEM) to determine their high-resolution 3D structures. We vitrified aliquots of the purified ACs, and the best grids were used for CryoEM data acquisition using the parameters described in Table S1. Image processing and subsequent 3D reconstruction procedures are detailed in the Materials and Methods section and Figs. S6–S9. The 2D classification of particles of the four ACs revealed that whereas ACI and AFCB formed heptameric rings, ACII and ACIII assembled into octameric rings, mirroring their modern counterparts (Figs. 2–6). Regarding their overall structure, a typical chaperonin double-ring structure was observed for ACIII and AFCB. By contrast, ACI showed single-ring structures, and ACII displayed a mixture of double rings (majority) and single rings.

Detailed 3D analysis of ACI confirmed its assembly into stable, single heptameric rings (Fig. 2a and Figure S6). While the “open state” structure provided valuable insights, its resolution was likely limited by the inherent structural flexibility of the chaperonin, particularly in the apical domain. This flexibility is evident in the lidless ACI map (Fig. 2a and Figure S6), which shows well-resolved α-helices in the equatorial domain but less defined features in the apical and intermediate domains (EMD-54342). Although these regions are typically harder to resolve, the increased dynamics of the apical domains might have been crucial in the early evolution of Group I chaperonins.

To further investigate the ACI single-ring architecture, we compared its sequence and structure with the well-characterized *E. coli* single-ring mutant (SR1) (Chatellier et al. 2000). Four amino acid substitutions (R452E, E461A, S463A, and V464A) distinguish SR1 from canonical *E. coli* GroEL and are known to disrupt double-ring assembly (Chatellier et al. 2000). In ACI, three functionally analogous substitutions are present (E460R, S463A, and V464E), which, although less drastic than in SR1, are also located at the ring–ring interface and are likely to interfere with the inter-subunit interactions required for double-ring formation (Fig. 2c) (Note that residue numbering corresponds to the respective organism's sequence, being R452 in *E. coli* and R460 in ACI). Ancestral sequence reconstruction for these four residues yielded high posterior probabilities: R (0.89), D (0.95), A (0.82), and E (0.48). Their evolutionary trajectories further highlighted dynamic shifts (Table S2): the probability of residue R increases from 0.89 to 1 by node 121, indicating fixation; Residue D, initially highly probable (0.95), decreases to 0.75 at node 108 and is gradually replaced by E from node 113, reaching fixation by node 128; Residue A decreases in probability to 0.44 at node 104 and is then replaced by S from node 109, becoming fixed by node 121; Residue E increases in probability up to node 107 but is ultimately replaced by V with probability 1 at node 108.

ACII was purified as a mixture of monomers and oligomers, and the presence of ATP triggered the typical conformational changes in chaperonins, transitioning between open and closed states. The 2D and 3D analysis of the particles revealed both single- and double-ring structures in both open and closed configurations **(**Fig. 3b and Fig. S7). However, only the closed double-ring structure, the most abundant oligomeric state (35% of all particles), yielded a map with sufficient resolution for atomic modeling (Fig. S7c) (PDB: 9RWP). When comparing this structure with modern chaperonins such as *E. coli* GroEL (Group I) or the group II eukaryotic chaperonin CCT (Fig. 4), we found that ACII possesses a built-in helical lid protrusion, similar to CCT. This feature allows ACII to close its cavity without needing a separate co-chaperonin like GroES, which GroEL requires. This structural element suggests that ACII developed an early mechanism for cavity closure. This mechanism is independent of a GroES-like co-chaperonin and predates the more complex mechanisms seen in modern Group II chaperonins, with CCT being the most complex example (Fig. 4b).

An interesting observation was that the closed conformation of ACII is less tightly locked than that of CCT (compare central and right structures in Fig. 4a, respectively), indicating distinct closure mechanisms between the two chaperonins. To understand the atomic-level changes that drive CCT-like closure, we first analyzed the structural changes observed within the apical, intermediate, and equatorial domains of ACII and CCT subunits. By calculating the root-square-mean deviation, which measures differences in protein backbones, we found that the main changes occur in the arrangement of the apical region, including the intermediate domain (Fig. 4b). Further examination of the intra-subunit interactions highlighted several notable aspects. In the built-in lid, CCT contacting-subunits exhibited somewhat different interactions. For example, a salt bridge forms between H312 of CCT5 (the CCT subunit most similar to ACII in the apical region) and E330 of CCT2. These interactions are further stabilized by hydrogen bonds between H312 and Q316 of CCT5 with T334 and E330 of CCT2, respectively (Fig. 4c). In ACII, a similar salt bridge exists between H299 and E334. However, due to a lack of interactions in the tip of the apical region, E334 forms a stronger interaction with K303. The positive charge of K303 allows for an additional interaction, similar to that observed with Q316 of CCT5 (Fig. 4c). In the central part of the helical protrusion, CCT forms strong interactions between K282 of CCT5 and D336 of CCT2, generating a salt bridge in the middle of the helix. At the end of the protrusion, these interactions are reinforced by the hydroxyl group of Y274 (CCT5), which interacts with K274 and E271 of CCT2. In ACII, these interactions are absent. Only a hydrogen bond is formed by P258 and Q267 (Fig. 4c, left). Additionally, potential repulsive forces along the helical protrusion in ACII further support this (Fig. 4c, right). Altogether, these observations suggest that while ACII possesses a built-in lid that reduces reliance on a GroES-type cochaperonin for cavity closure, the resulting closure is less complete than that of CCT subunits, where total sealing prevents solute entry or exit.

In the case of ACIII, oligomeric structures were visible in the absence of ATP, and they all formed double-ring structures (Fig. S8). The structure obtained (Fig. 5) (PDB: 9RWQ) resembles that of the modern thermosomes (Chaston et al. 2016) (Fig. S1), although the flexibility observed in the apical domains (Fig. S8c) prevented us from modeling these regions (Fig. 5b).

Finally, our structural characterization of AFCB (Ancestral FCB Chaperonin) revealed a strikingly modern architecture (Fig. 6 and Fig. S9). There was no need for the addition of ATP to generate a stable, homogeneous population of double-ring oligomers (Figure S9a and b). This enabled us to obtain a 3.4 Å resolution map (Fig. 6a) and build the corresponding atomic model (Fig. 6c) (PDB: 9RWR). Unsurprisingly, AFCB closely resembles *E. coli* GroEL, sharing a 71% sequence identity (see Fig. 6b). Although resurrecting the GroES-type cochaperonin was beyond the scope of this study, an analysis of key residues involved in the GroEL-GroES interaction showed that all three essential residues are conserved in AFCB (Fig. 6d). This strongly suggests that AFCB likely interacts with a GroES-type cochaperonin, hinting at the co-evolution of the chaperonin and its cochaperonin counterpart within the FCB lineage.

### Functional Evaluation of Ancestral Chaperonins

**Fig. 7.**
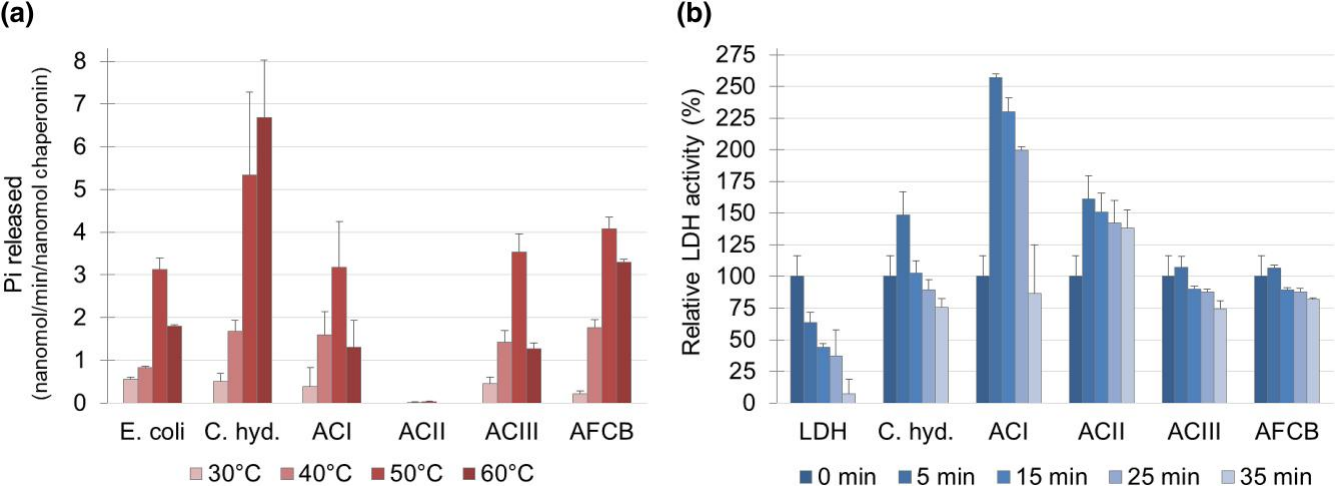
Chaperonin activity assays. **a**) ATP hydrolysis activity: Average ± SD (*n* = 3) phosphate released by chaperonins at 30 °C, 40 °C, 50 °C, and 60 °C, normalized to control without chaperonin. **b**) Thermal protection assay: Average ± SD (*n* = 2) activity of lactate dehydrogenase (LDH) after heat stress (48 °C) in the presence of ancestral (ACI, ACII, ACIII, AFCB) and modern (*E. coli*—*Escherichia coli*, C. hyd.—*Carboxydothermus hydrogenoformans*) chaperonins, normalized to the activity of LDH alone before heat stress.

We next compared the activity of ACs with that of modern *E. coli* (Group I, Uniprot: P0A6F5) and *Carboxydothermus hydrogenoformans* (Group III, UniProt: Q3AF10), expressed and purified under identical conditions. ATPase activity was monitored in 10 °C increments from 30 °C and 60 °C using BIOMOL GREEN Reagent, which detects phosphate released during ATP hydrolysis (see Materials Methods) (Geladopoulos et al. 1991) (Fig. 7a). The Group III chaperonin from *C. hydrogenoformans*, a thermophile, showed the highest rate of ATP hydrolysis, followed by AFCB. The remaining chaperonins *E. coli* GroEL, ACI and ACIII exhibited similar hydrolysis patterns. *C. hydrogenoformans* ATPase hydrolysis peaked at 60 °C, while the others peaked at 50 °C. No ATP hydrolysis was detected for ACII. ATPase activity for *C. hydrogenoformans* and *E. coli* was consistent with previous reports (Mendoza et al. 1996; An et al. 2017).

To evaluate chaperonin function, we assessed their ability to protect lactate dehydrogenase (LDH) from heat-induced inactivation. LDH catalyzes the conversion of pyruvate to lactate while reducing NAD+ to NADH, which can be monitored spectrophotometrically at 340 nm. When incubated at 48 °C, LDH lost 50% of its activity within 15 min and almost all activity by 35 min. However, LDH activity was preserved in the presence of ancestral or modern chaperonins (Fig. 7b).

The modern *C. hydrogenoformans* chaperonin initially boosted LDH activity after 5 min, followed by a return to baseline levels at 15 min and a slight decrease to 75% of the initial activity by 35 min. AFCB and ACIII showed similar patterns, with a small initial increase in LDH activity before stabilizing at ∼75% activity after 35 min. By contrast, ACI and ACII provided sustained protection: LDH activity remained at ∼150% with ACII throughout the experiment, while ACI caused a 2.5-fold increase in activity within the first 5 min before returning to baseline levels by the end of the incubation.

### Evolutionary Trends in Electrostatic Potential and Folding Energy

**Fig. 8.**
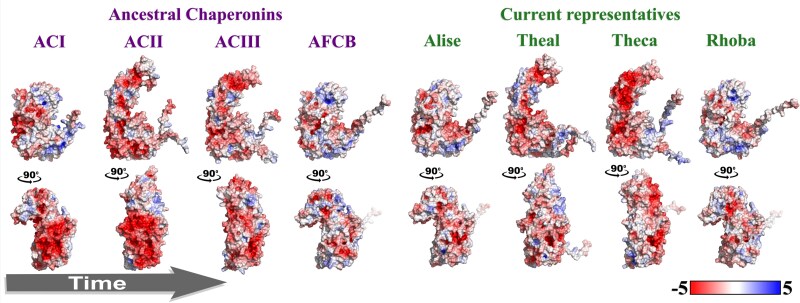
Structural and electrostatic potential of ancestral chaperonins. The distribution of the electrostatic potentials calculated with the APBS method, implemented in PyMol (http://www.pymol.org/pymol), on the molecular surface of AlphaFold3-generated structural models of ACI, ACII, ACIII and AFCB ancestral chaperonins together with examples of current representatives of each group (see Table 1): chaperonin GroEL from *Alicyclobacillus sendaiensis* (Alise), thermosome subunit beta from *Thermococcus alcaliphilus* (Theal), TCP-1/cpn60 chaperonin from *Thermosinus carboxydivorans* (Theca), and 60 kDa chaperonin from *Rhodothermaceae bacterium* (Rhoba). Color-coding scale ranges from most negative potential (red) to most positive potential (blue).

To further characterize ACs and identify potential features, we analyzed their electrostatic properties using the APBS method (Jurrus et al. 2018) (implemented in PyMol), and calculated variations in folding energies with FoldX (see Materials and Methods) (Table 2). Electrostatic map calculations revealed that the oldest reconstructed ancestors (ACI and ACII) predominantly displayed a negative surface electrostatic potential (Fig. 8).

**Table 2.** Correlation between folding energies and ancestrality, for each group

Group I	Group II	Group III	FoldX energy terms
−0.44	0.05	▴ 0.54	Total stability
▴ 0.94	▴ 0.90	0.43	Backbone hydrogen bonds
▴ 0.77	▴ 0.54	−0.17	Side-chain hydrogen bonds
0.11	0.46	▴ 0.63	Van der Waals
0.13	▴ 0.70	−0.26	Electrostatic (charge-charge)
0.11	−0.13	▾−0.52	Polar solvation
−0.07	0.47	▴ 0.55	Nonpolar solvation
0.41	−0.47	0.15	Van der Waals clashes
−0.42	▾−0.71	−0.44	Side-chain entropy
▾−0.86	▾−0.50	−0.41	Main-chain entropy
▾−0.60	−0.47	▴ 0.86	Torsion energy
−0.25	0.03	▾−0.51	Backbone Van der Waals clashes
0.12	−0.29	▾−0.68	Helix dipole effects
▴ 0.59	▾−0.51	0.41	Ionization energy

▴—significant positive correlation (>0.50).

▾—negative significant correlation (<−0.50).

Computational analysis of free folding energies revealed significant correlations between ancestrality and key structural parameters—hydrogen bonds, entropy, electrostatic interactions, solvation effects, Van der Waals interactions, and torsion energy—all of which contribute to protein stability (Table 2). Group I ancestors showed significant positive (≥0.5) correlations with backbone hydrogen bonds (*r* = 0.94), side-chain hydrogen bonds (*r* = 0.77), and ionization energy (*r* = 0.59); and significant negative (≤−0.5) correlations with main-chain entropy (*r* = −0.86) and torsion energy (*r* = −0.60). Group II ancestors showed significant positive correlations with backbone hydrogen bonds (*r* = 0.90), side-chain hydrogen bonds (*r* = 0.54) and charge-charge interaction (*r* = 0.70); and significant negative correlations with side− (*r* = −0.71) and main-chain (*r* = −0.50) entropy, and ionization energy (*r* = −0.51). Group III ancestors showed significant positive correlations with total stability (*r* = 0.54), Van der Waals interaction (*r* = 0.63), nonpolar solvation (*r* = 0.52), and torsion energy (*r* = 0.86); and significant negative correlations with polar solvation (*r* = −0.55), backbone Van der Waals clashes (*r* = −0.51), and helix dipole effects (*r* = −0.68). These divergences suggest that different chaperonin lineages developed specialized strategies, using diverse physicochemical mechanisms, to optimize protein stability.

## Discussion

### Intermediate Single-ring Structures in Ancestral Chaperonins Support a Stepwise Oligomerization Model

Our phylogenetic analysis, based on 96 modern sequences, produced a topology consistent with previous studies (Gupta 2004; Cavalier-Smith 2006; Ward et al. 2019; Cavalier-Smith and Chao 2020; Rebeaud et al. 2021) and allowed us to resurrect and characterize four ancestral nodes (ACI, ACII, ACIII, and AFCB) (Figs. S2 and S3). We observed that the earliest ancestors—ACI (bacterial GroEL) and ACII (archaeal thermosome)—are single-ring structures. In Group I systems, double-ring structures are known to dissociate upon ATP hydrolysis, increasing folding capacity for larger substrates, and single-ring structures have been observed in bacteriophage-encoded chaperonins (eg ϕ-EL and OBP), the human mitochondrial chaperonin, and bacterial GroEL/ES systems (Bhatt et al. 2018). To our knowledge, ACII represents the first reported single-ring structure for a Group II chaperonin.

The evolutionary progression from an exclusively single-ring structure in ACI, to a mix of single and double rings in ACII, and finally to an exclusively double-ring assembly in ACIII (Fig. 1), suggests a gradual, step-by-step evolution from single- to double-ring chaperonins. This pattern supports a timeline where ACI is the oldest ancestor, followed by ACII and then ACIII—with the latter likely arising through horizontal gene transfer between an ancestral archaeon and a Firmicutes bacterium (Techtmann and Robb 2010; Rebeaud et al. 2021) rather than being a direct descendent from LUCA (An et al. 2017). Sequence divergence patterns (Table 1) further corroborate this timeline, showing ACI as the most divergent, followed by ACII, ACIII, and finally AFCB.

Structurally, ACII features a unique build-in helical protrusion that acts as a lid, eliminating the need for a GroES-type cochaperonin for cavity closure (Fig. S7). This feature may reflect an intermediate evolutionary stage between the architectures of modern Group I and II chaperonins. Another key difference lies in their subunit structure: ACI has seven subunits while ACII has eight—a stoichiometry that mirrors their modern counterparts (Figs. 2 and 3). These differences could stem from the presence of multiple assembly types in LUCA, or reflect lineage-specific changes in ring size over evolutionary time. Such evolutionary divergence in subunit stoichiometry is also observed in modern complexes: for example, archaeal peroxiredoxins (a thiol-dependent peroxidase that protects cells from oxidative damage) often form pentameric rings, whereas bacterial homologs adopt hexameric forms (Himiyama et al. 2019; Stroobants et al. 2019).

Functionally, chaperonins operate through two major conformational states: an open state for substrate recognition, and a closed state that traps and facilitates protein folding. The ACs appear to have developed two distinct mechanisms for cavity closure. Bacterial chaperonins co-evolved with a small capping oligomer (the cochaperonin), whereas archaeal and eukaryotic chaperonins developed an extra sequence, a lid-like protrusion that fulfils the same locking role. These functional variations suggest that the conserved folding chamber of chaperonins adapted to distinct functional and environmental pressures. While the evolutionary relationships between Bacteria, Archaea, and Eukarya are still debated (and may never be fully clarified), our findings align with the classical model in which Bacteria are evolutionarily closer to LUCA (Gribaldo and Brochier-Armanet 2006).

The evolution of protein complexity—from simpler to more structurally and functionally elaborate proteins (such as from monomers to oligomers or through the acquisition of additional domains)—has been explored using ASR in previous works (Hochberg and Thornton 2017). In particular, multimerization has been investigated in the ATPase complex (Finnigan et al. 2012) and RuBisCO (Schulz et al. 2022; Amritkar et al. 2025) protein families. Thornton and colleagues have shown that such transitions can occur naturally through just one or a few mutations (Hochberg and Thornton 2017; Pillai et al. 2022). Building on this work, our study reveals an evolutionary trajectory within the chaperonin family. We found that ACI likely transitions between monomeric and single-ring states, representing an early stage in oligomerization. By contrast, ACII and ACIII predominantly form stable double-ring assemblies, suggesting that the molecular framework for full oligomeric complexity was already established in these groups. The coexistence of both single- and double-ring assemblies in ACII illustrates an evolutionary intermediate in which the molecular architecture for oligomerization had emerged but was not yet fully optimized—likely lacking one or a few key mutations needed to substantially enhance binding affinity and stabilize the double-ring state (Hochberg and Thornton 2017; Pillai et al. 2022). While we did not explicitly test mechanistic models for the evolution of molecular complexity, our analysis of the posterior probabilities of the amino acids at positions known to disrupt double-ring assembly (R452E, E461A, S463A, V464A) in *E. coli* GroEL (Chatellier et al. 2000) showed that the ancestral sequence (ACI) retained three of these substitutions with high confidence (Table S2). Tracking these probabilities across nodes revealed dynamic shifts: residues that potentially prevent double-ring formation in ACI gradually gave way to substitutions that favor stable double-ring interfaces. Together, these patterns further support a gradual evolutionary transition from a single-ring to a double-ring architecture in early Group I chaperonins—a shift predicted to have reached completion by node 128 (Fig. S3), corresponding to the divergence time of the FCB group.

### ATP-dependent Stability and Activity in Ancestral Chaperonins

ATP played an essential role in the assembly of ACI oligomers and often enhanced the assembly of ACII by increasing the number of oligomers. By contrast, ACIII and AFCB did not require ATP for either assembly or stability. This suggests that ATP dependency was strongest in the most ancestral nodes, particularly ACI. Some modern thermosomes exhibit ATP-dependent oligomer formation, which could be an evolutionary remnant from ancestral times (Chaston et al. 2016).

Under neutral conditions, ATPase activity was detected in all ancestral chaperonins tested, with the exception of ACII (Fig. 7a). The absence of detectable activity in ACII could indicate a requirement for specific cofactors, such as ammonium salts, as observed in certain methanogenic archaea (Andra et al. 1998) and bacterial thermosome-like systems (Techtmann and Robb 2010).

Despite its lack of detectable ATPase activity, ACII proved to be highly effective at protecting LDH from heat inactivation (Fig. 7b). Indeed, it outperformed the other chaperonins in maintaining LDH stability at 48 °C, sustaining 150% of its activity throughout the experiment. Although we initially attempted to assess the ability of these ACs to assist in refolding of chemically denatured proteins, technical challenges prevented these assays from being carried out. Specifically, we were unable to detect successful refolding even with *E. coli* GroEL as a control, likely due to the absence of its essential co-chaperonin GroES under our experimental setup. Consequently, we shifted our focus to a more limited, but still insightful, protective assay (LDH heat inactivation), which provided valuable insights into the stress-response capacity of the ACs. While molecular crowding could theoretically explain this effect, the variations seen among the different chaperonins suggest it was not the primary cause.

Although the closest modern sequences to our reconstructions (in the NCBI nr-database) were from thermophilic organisms (Table 1), the activity of our ancestral proteins was comparable with that of the mesophile GroEL from *E. coli* (Mendoza et al. 1996). This contrasts with other proteins reconstructed through ASR, which often display high thermostability (Wheeler et al. 2016). This suggests that ACs might be a unique case where extreme thermal stability was not a defining ancestral trait or a functional requirement. However, our results on ATP-dependent stability and activity do not necessarily rule out thermal adaptation. Some modern thermophilic chaperonins also exhibit ATP-dependent oligomerization, and ATPase activity alone may not fully reflect thermal robustness (Andra et al. 1998; Techtmann and Robb 2010; Chaston et al. 2016).

### Predicted Electrostatic Features Suggest Ancient Environmental Pressures

The electrostatic potential on the surface of a protein is crucial for molecular interactions, and changes in this potential can indicate how proteins adapt to environmental pressures. Our analysis revealed that ACI and ACII, likely the earliest ancestral proteins, have highly negative surface charges (Fig. 8). This suggests that ACs in Groups I and II evolved in conditions where negative surface charges were advantageous. For example, these changes could have helped stabilize proteins in acidic or high-salt conditions (Reed et al. 2013), or increased thermal stability at high temperatures (Vieille and Zeikus 2001). Interestingly, the modern homolog with the highest sequence identity to ACI is an acidophilic thermophile (Table 1) (Tsuruoka et al. 2003), echoing the adaptation of ancestral thioredoxins to acidic environments (Perez-Jimenez et al. 2011). Given the proposed evolutionary link between chaperonins and thioredoxins (Dekker et al. 2011; Rowland and Robb 2017), this parallel supports the idea that both families may have originated in harsh primordial environments, where electrostatic surface properties were crucial for protein stability. Specifically, positive surface charges could have counteracted proton-driven destabilization in low-pH conditions, while surface charges might have been essential in maintaining solubility and preventing aggregation in high-salinity habitats (Reed et al. 2013). As environmental conditions fluctuated over evolutionary time, increasing positive charges may have been selectively favored to balance protein stability and function, highlighting their adaptive versatility. While the electrostatic profiles and modern homologs suggest that ACs may have been adapted to high-temperature, low-pH, or high-salinity environments, these hypotheses remain speculative. We did not experimentally test activity under extreme conditions, and so direct validation of these proposed environmental adaptations will require further research.

### Energetic Trends and Environmental Adaptations of Ancestral Chaperonins

Trends in energy variation reveal distinct evolutionary paths among the three chaperonin groups, driven by environmental pressures (Table 2). In Groups I and II, overall stability remained relatively constant. However, we observed a significant increase in hydrogen bonds and a decrease in the entropy term (calculated by FoldX encompassing main-chain and side-chain conformational entropy) (Schymkowitz et al. 2005; Delgado et al. 2025). This suggests that ACs in these groups could have evolved more ordered and rigid structures, to maintain stability under stress. This pattern aligns with observations in thermophilic proteins, which enhance stability through stronger intramolecular forces rather than significant changes in global stability (Vieille and Zeikus 2001).

In Group II, the inferred increase in charge-charge interactions and decrease in ionization energy point to adaptations to high-salt environments, where balancing electrostatic interactions is crucial for solubility and function. Similar adaptations are found in halophilic proteins, which optimize surface charges to prevent aggregation in saline conditions (Madern et al. 2000). Conversely, the increasing ionization energy in Group I suggests adaptations to acidic environments, where stabilizing charged residues is essential for maintaining structural integrity—a common characteristic of acidophilic proteins (Garcia-Moreno 2009).

For Group III ancestors, a notable increase in total stability appears to be driven by strengthened Van der Waals forces and reduced backbone clashes, indicating enhanced hydrophobic packing. This is a well-documented adaptation in thermophilic proteins, where compact hydrophobic cores provide resilience to high temperatures (Vieille and Zeikus 2001; Reed et al. 2013). Additionally, a decrease in polar solvation and increase in nonpolar solvation suggest reduced hydrophilicity. This could reflect adaptations to low-water-activity environments, such as hypersaline, acidic, or thermophilic habitats (Vieille and Zeikus 2001; Tadeo et al. 2009; Kraml et al. 2019; Benison et al. 2021).

Together, these trends suggest that environmental pressures were a significant factor in shaping the evolution of chaperonins, driving structural adaptations to extreme conditions. This supports the hypothesis that early chaperonins likely evolved in halo-acidophilic, possibly thermophilic environments, similar to those thought to exist on early Earth (Sugisaki et al. 1995), gradually adapting as Earth's environment became more neutral over time. This perspective differs from previous analyses of ancestral nucleoside diphosphate kinases and ribosomal protein uS8s, which proposed a primitive alkaline habitat (Fujikawa et al. 2024). However, these scenarios are not mutually exclusive, and the selective pressures that shaped one protein family may have differed from those acting on another. Nonetheless, previous (Kitadai and Maruyama 2018) and recent (Šponer et al. 2024) advances in prebiotic chemistry support the idea that life could have formed in acidic hydrothermal environments. This notion is reinforced by studies on ancestral thioredoxins (Perez-Jimenez et al. 2011), which add to the idea that early life flourished in hot, acidic conditions. These remain open questions, inviting further research.

Although these structural trends might suggest adaptations to extreme environments, we recognize that our experimental support for this hypothesis is limited, and computational estimates given by programs such as FoldX have known limitations and should be interpreted with caution (Buß et al. 2018). Furthermore, ATPase activity assays suggest that the reconstructed proteins exhibit limited activity at temperatures above 50 °C, although these two properties are not necessarily correlated. To gain a more complete understanding of the environmental preferences of these ancestral proteins, future studies should examine a broader range of physicochemical conditions, including low pH and high salinity.

## Concluding Remarks

This study demonstrates that ASR, despite its inherent challenges and the practical limitations of our own implementation, can reveal unexpected structural features in ACs. Our findings support a model where chaperonins evolved gradually from single-ring to double-ring assemblies, with ACII potentially representing an evolutionary intermediate. These structural changes, coupled with shifts in ATP dependence and stability, suggest an overall trend toward greater molecular complexity (Pillai et al. 2022).

Although the energetic patterns we describe were inferred computationally and rely on structural models rather than direct measurements—thus requiring cautious interpretation—they nonetheless indicate adaptations to acidic, high-salinity environments. These environmental pressures may have shaped early protein evolution (Sugisaki et al. 1995). While some of these insights remain to be experimentally validated, they align with findings from their closely related thioredoxin family (Dekker et al. 2011; Perez-Jimenez et al. 2011; Rowland and Robb 2017).

Overall, our work contributes to the growing evidence on how complexity emerges in molecular systems. It also highlights evolutionary patterns that could guide future research into key transitions in early cellular life and the development of modern protein architectures, particularly within the chaperonin family.

## Materials and Methods

### Phylogenetic Analysis and Ancestral Sequence Reconstruction

The chaperonin GroEL sequence from *E. coli* (UniProt: P0A6F5) was used as query in a blast search on the Kyoto Encyclopedia of Genes and Genomes server (https://www.genome.jp/tools/blast). Searches were performed individually for major taxonomic groups to ensure broad representation, and hits were filtered using a similarity threshold (10^−6^) (Kanehisa and Goto 2000). The resulting FASTA sequences were downloaded and curated to generate a manageable and taxonomically balanced dataset for ancestral sequence reconstruction. We selected 96 sequences based on the following criteria: (i) broad taxonomic coverage, (ii) sequence completeness and quality—excluding partial or poorly annotated entries—and (iii) removal of redundancy using CD-HIT (https://hcc.unl.edu/docs/applications/app_specific/bioinformatics_tools/removing_detecting_redundant_sequences/cd_hit/) (Huang et al. 2010). Multiple sequence alignment was performed with MAFFT (L-INS-i strategy; https://mafft.cbrc.jp/alignment/server) (Katoh et al. 2019), followed by manual inspection and curation. Insertions and deletions (*indels*) were carefully reviewed using a conservative approach: clade-specific *indels* were retained, while individual or sporadic *indels* were removed. This strategy aimed to reduce alignment noise while preserving phylogenetically informative features. We note that sequence selection and *indel* treatment can significantly influence phylogenetic tree topology and ancestral sequence inference. Although we did not compare results across different, independently curated, datasets, our curation strategy was designed to reflect the best practices in the field, as recommended in previous studies (Ross et al. 2022).

ProtTest 3.4 was used to estimate a model of amino acid substitutions (Darriba et al. 2011). The model chosen, according to the Akaike information criterion, was the Le Gascuel 2008 model, with gamma distribution and a proportion of invariable sites (LG + I + G model) (Le and Gascuel 2008). The phylogenetic history of the 96 chaperonin sequences was reconstructed using the Bayesian method, implemented in MrBayes 3.2.7 (Hall 2006). The analysis used two independent Markov-chain Monte Carlo runs, each with two chains, performed over 800,000 generations until convergence was obtained, which was confirmed by the following indicators: (i) the standard deviation of split frequencies was less than 0.01; (ii) stationarity was checked by plotting likelihood scores (lnL) against a number of generations; and (iii) for each parameter in the model, a value of 1.000 for the potential scale reduction factor was obtained. Trees were sampled every 100 generations. Each run produced 8,501 trees, from each 6,376 were included, totaling 12,752 trees from which a consensus tree with high probabilities at the nodes was obtained. Marginal reconstruction of ancestral sequences was done with PAML 4.9 CodeML, using the LG substitution model with gamma-distributed rates across sites (Yang 1997; Le and Gascuel 2008). The most probable ancestral sequences of the four nodes were initially inferred by PAML, which computes over the entire alignment, including positions with gaps that characterize the different groups. To refine these sequences, and whenever needed, residues were replaced with gaps from the closest modern sequence (defined by short branch length), producing ancestral sequences aligned in length with the modern homologs (Boucher et al. 2014).

The sequences (Table S3) corresponded to the last common ancestor nodes of Group I (ACI, for ancestor group I, aged ∼4.1 to 3.6 Ga), Group II (ACII, ∼4.2 to 3.8 Ga), Group III (ACIII, divergence time approximately 3.0 to 2.5 Ga corresponding to divergence times of Firmicutes and Cyanobacteria), and the last common ancestor of FCB Group (AFCB, ∼3.0 to 2.6 Ga). Divergence times were gathered from various sources and synthesized using the TimeTree project (Kumar et al. 2022). Finally, we tested alternative rooting strategies, either between Group I and Groups II + III or between Groups I + II and Group III, but since the resulting ancestral sequences showed >96% identity across all nodes, and given current evidence supporting Group III as an archaeal derivative, we rooted the tree between Group I (Bacteria) and Group II (Archaea).

### Cloning, Expression and Purification of Ancestral Enzymes

The amino acid sequence of each protein included a C-terminal His-tag and was translated into the corresponding DNA nucleotide sequence, codon-optimized for *E. coli* using the OptimumGene Codon Optimization Analysis and Algorithm (GenScript Biotech, Rijswijk, Netherlands). The optimized genes were synthesized by the GenScript Recombinant Protein Expression Service (GenScript Biotech) and ligated into plasmid pET-30a(+) using the CloneEZ method. Plasmids were cloned, amplified in *E. coli* DH5α (DE3), and purified from kanamycin-resistant cells using the QIAprep Miniprep Kit (Qiagen GmbH, Hilden, Germany). The integrity of the plasmid preparations was verified by agarose gel electrophoresis. *E. coli* BL21 (DE3) cells were transformed with the purified plasmids and selected by kanamycin resistance. Recombinant protein expression was induced with 1 mM IPTG for 4 h at 37 °C. After centrifugation, the cell pellet was resuspended in nickel nitrilotriacetic acid (Ni-NTA) binding buffer (NiBB) (20 mM 4-(2-hydroxyethyl)-1-piperazineethanesulfonic acid [HEPES] pH 7.4, 20 mM imidazole, 500 mM KCl, 1 mM DTT and 15% glycerol [v/v]) supplemented with a protease inhibitor cocktail (Roche Holding AG, Basel, Switzerland). The resuspended solution was sonicated three times for 1 min each time (cycles of 10 sec *on*, 50 sec *off*), with an amplitude of 50% in a Branson Digital Sonifier 250, and centrifuged at 15.000 × g in a Beckman Coulter ultracentrifuge with a Type 50.2 Ti rotor (40 min at 4 °C). The supernatant was filtered (0.45 μm) and loaded onto a Ni-NTA agarose column (HisTrap FF 5 ml, GE Healthcare, Chicago, IL) using a fast protein liquid chromatography (FPLC) apparatus (GE Healthcare). The loaded column was then washed with five column volumes of NiBB and eluted with Ni-NTA elution buffer (NiEB) (same as NiBB but with 500 mM imidazole) using a gradient of 50% over 15 min. Fractions (2 ml) were collected and analyzed by SDS-PAGE, and those containing the corresponding protein were pooled and concentrated by centrifugation (4.000 × *g*, in a Hitachi centrifuge with a R15A rotor, at 4 °C) using 50 kDa or 100 kDa cut-off Amicon Ultra 15 filters (Merck Millipore, Darmstadt Germany). The concentrated fractions containing the protein of interest were loaded onto size exclusion chromatography (SEC) Superose 6 Increase 10/300 GL (GE Healthcare) columns, previously equilibrated in SEC buffer (SECB) (20 mM HEPES pH 7.4, 150 mM KCl, 10% glycerol [v/v]). Proteins were eluted in SECB and fractions (0.5 ml) were analyzed by SDS-PAGE and by negative staining electron microscopy. When necessary, complex formation was induced with 25 mM MgCl_2_ and 5 mM ATP (Chaston et al. 2016). Fractions with proteins forming the typical ring complex were supplemented with 5% glycerol, snap frozen in liquid nitrogen and stored at −20 °C.

### ATPase Activity Assay

The ATP hydrolysis rates of ancestral chaperonins (ACI, ACII, ACIII, and AFCB) and *C. hydrogenoformans* (UniProt: Q3AF10) were calculated as the amount of phosphate (Pi) released (nanomol/min/nanomol chaperonin) based on the theoretical molecular weight of each oligomeric complex. The theoretical molecular weight was determined from the protein amino acid sequence using ProtParam (https://web.expasy.org/protparam/). A single-ring oligomer was assumed for ACI, whereas a double-ring oligomer was assumed for the other chaperonins. Chaperonin proteins at concentrations of 0.35 µg µl^−1^ or 0.5 µg µl^−1^ were incubated in 50 µl of buffer containing 50 mM HEPES pH 7.4, 150 mM KCl, 5 mM MgCl_2_ and 0.5 mM ATP. Reactions without chaperonin served as negative controls, and subtracted from the results. Incubations were carried out on a thermoblock with gentle agitation (300 rpm) across a range of temperatures (30 °C, 40 °C, 50 °C, and 60 °C). The reaction was stopped with BIOMOL GREEN™ Reagent. Following a 20-min incubation at room temperature (RT), absorbance at 650 nm was measured using a SpectraMax iD3 spectrophotometer. The amount of phosphate released was estimated using a standard curve generated from phosphate dilutions ranging from 2 to 0 nmol.

### Chaperone Activity Assay With Lactate Dehydrogenase

The chaperone activity of the resurrected chaperonins was assessed as described (Hristozova et al. 2016), with minor modifications. The assay measures the ability of each chaperonin to preserve LDH activity under thermal stress. Enzyme inactivation was induced at 48 °C in 50 µl of HEPES buffer (50 mM HEPES, pH 7.4, 150 mM KCl, 5 mM MgCl_2_) containing 0.1 µM LDH, 0.4 µg µl^−1^ chaperonin, and 1 mM ATP. Reactions without chaperonin served as negative controls. Incubations were performed on a thermoblock with gentle agitation (300 rpm). At each time point, 5 µl of the incubation mixture was transferred to a cuvette containing 495 µl of assay buffer (20 mM HEPES, pH 7.4, 50 mM KCl, 0.3 mM NADH, and 1.6 mM sodium pyruvate). The cuvette was immediately sealed with parafilm and mixed by inversion. The decrease in absorbance at 340 nm, reflecting NADH consumption, was monitored for 3 min at RT, with readings taken every 15 s. All assays were performed in duplicate. The slope of each reaction was calculated using Microsoft Excel, and the mean and standard deviation were determined for each sample. Results were normalized to the enzyme activity prior to thermal stress.

### Sample Preparation for Cryogenic Electron Microscopy

Samples were vitrified in a Vitrobot Mark IV (FEI, Eindhoven, Netherlands) at 4 °C and 100% humidity. A drop of 3 μl of sample was absorbed onto Quantifoil R 2/2 300 mesh and glow-discharged (25 mA, 15 s) grids. The force and blotting time were −2 and 2 s, respectively. Vitrified grids were stored in liquid nitrogen before screening. The vitrified samples were checked, and data from the best sample was acquired in a 200 kV FEI Talos Arctica equipped with a Falcon III direct electron detector at the Spanish National Center for Biotechnology CryoEM facility (CryoEM CNB-CSIC). The images of last common ancestors of Group I and Group III (ACI, ACIII) proteins were acquired at a nominal magnification of ×120,000 (corresponding to a pixel size of 0.85 Å/pixel) and the AFCB Group images at ×73,000 (corresponding to a pixel size of 1.42 Å/pixel) with a defocus range of −1.2 to −3.0 μm in all samples. The ACII protein was first checked on a 200 kV FEI Talos Arctica at the CNB followed by data acquisition on a FEI Titan Krios electron microscope operated at 300 kV, equipped with a Gatan Quantum K3 Summit direct electron detector at Diamond Light Source (Oxford, UK). Data collection was carried out with a × 130,000 nominal magnification (yielding a pixel size of 0.921 Å/pixel) and a defocus range of −1.0 to −2.4 μm.

### Image Processing and Three-dimensional Reconstruction

Image processing of all samples was performed following a similar workflow. All programs used for image processing to obtain the different 3D maps were implemented in Scipion (de la Rosa-Trevín et al. 2016). First, the movies were aligned using MotionCor (Zheng et al. 2017) and the outputs were subjected to CTF determination using Gctf (Zhang 2016). Particles were automatically picked with Xmipp3 (Vargas et al. 2013) and were subjected to several 2D classifications using Relion 2.0 (Fernandez-Leiro and Scheres 2017) and Cryosparc (Punjani et al. 2017) to exclude bad particles and ice contamination. Some of the more superior 2D classes were used as a template to generate an initial model using both CryoSPARC and RANSAC (Vargas et al. 2014). The initial models were low-pass filtered to 50 Å and used for 3D classifications. The classes with the best structural features were used for the reconstruction of the different chaperonins using Relion 5.0 and CryoSPARC, applying C7 and D8 symmetry for chaperonins ACI and ACII, respectively, and no symmetry (C1) for chaperonins ACIII and AFCB. The resulting maps were visualized with ChimeraX (Pettersen et al. 2021). The resolution of the final maps was estimated by the Fourier shell correlation method, with a cut-off of 0.143 and local resolution was calculated by Xmipp3-MonoRes (Vilas et al. 2018)

### Model Building

Models for each AC were generated with the AlphaFold3 server (Abramson et al. 2024) using the sequence of each chaperonin as a reference. The resulting models were docked into each cryo-EM density map by rigid body fitting with the Fit in Map tool of ChimeraX. Subsequently, the models were refined through multiple rounds of PHENIX 1.20.1-4487 (Adams et al. 2010) real-space refinement and manually adjusted using Coot 0.9.8.96 (Emsley et al. 2010) to improve structural restraints, including Ramachandran plot outliers, geometry, and rotamer restraints (Table S4). The restraints used in PHENIX real-space refinement included both standard restraints (bond, angle, planarity, chirality, dihedral, and nonbonded repulsion) and additional restraints (Ramachandran plot, C-beta deviations, rotamer, and secondary structure). The refinement process also combined morphing, simulated annealing, and rigid-body strategies.

A local grid search-based fit was incorporated into the refinement strategy to correct side-chain outliers, such as rotamers or regions with poor map fitting. Validation of the final models was done using the phenix.validation cryoem module in PHENIX.

### FoldX-based Stability Prediction of AlphaFold3 Models

Protein stability predictions were performed using FoldX 5.1 (Delgado et al. 2025) with AlphaFold3-generated structural models (Abramson et al. 2024) as input. The structural models used for these calculations correspond to the ancestral sequences at the nodes highlighted in Figure S3. The analysis was conducted *via* the command-line tool using the Stability function (foldx -c Stability –pdb), which calculates (i) the total energy (kcal/mol), reflecting overall structural stability; (ii) the Gibbs free energy change (ΔG, kcal/mol), representing the energy difference between folded and unfolded states; and (iii) individual energy contributions, including backbone hydrogen bonds, sidechain hydrogen bonds, Van der Waals clashes, electrostatics, and solvation energy (polar/apolar components). Default FoldX parameters (pH 7.0, 298 K, 0.05 M ionic strength) were applied without further refinement of the input models.

## Supplementary Material

msaf314_Supplementary_Data

## Data Availability

Cryo-EM data generated in this study have been deposited in the Electron Microscopy Data Bank under accession codes EMD-54342 for ACI, EMD-54339 for ACII closed-state double ring, EMD-54343 for ACII open-state double ring, EMD-54344 for ACII closed-state single ring, EMD-54345 for ACII open-state single ring, EMD-54340 for ACIII, and EMD-54341 for AFCB, respectively. The associated models have been also deposited in the Protein Data Bank under accession codes 9RWP for ACII closed state double ring, 9RWQ for ACIII and 9RWR for AFCB. All other data generated in this study are provided in the Information/Source Data file with this paper.
