# Supplementary material for: Ancestral Chaperonins Provide the First Structural Glimpse into Early Multimeric Protein Evolution
Source: Mol Biol Evol. 2025 Nov 28;42(12):msaf314. doi: 10.1093/molbev/msaf314 (PMC12696368; doi:10.1093/molbev/msaf314)
Supplement: msaf314_Supplementary_Data [file msaf314_supplementary_data.pdf]

# Supplementary Information

## Ancestral Chaperonins Provide the First Structural Glimpse into Early Multimeric Protein Evolution

Rita Severino[1,2], Jorge Cuéllar[3], Jorge Gutiérrez-Seijo[3], Moisés Maestro-López[3], Luis Sánchez-Pulido[1], César Santiago[3], Mercedes Moreno-Paz[1], José María Valpuesta[3,4] and Víctor Parro[1]

[1]Centro de Astrobiología (CAB), INTA-CSIC, Torrejón de Ardoz, Madrid, Spain

[2]University of Alcalá (UAH), Alcalá de Henares, Madrid, Spain

[3]National Center for Biotechnology (CNB), CSIC, Madrid, Spain

[4]Unidad de Nanobiotecnología, CNB-CSIC-IMDEA Nanociencia Associated Unit, Madrid, Spain

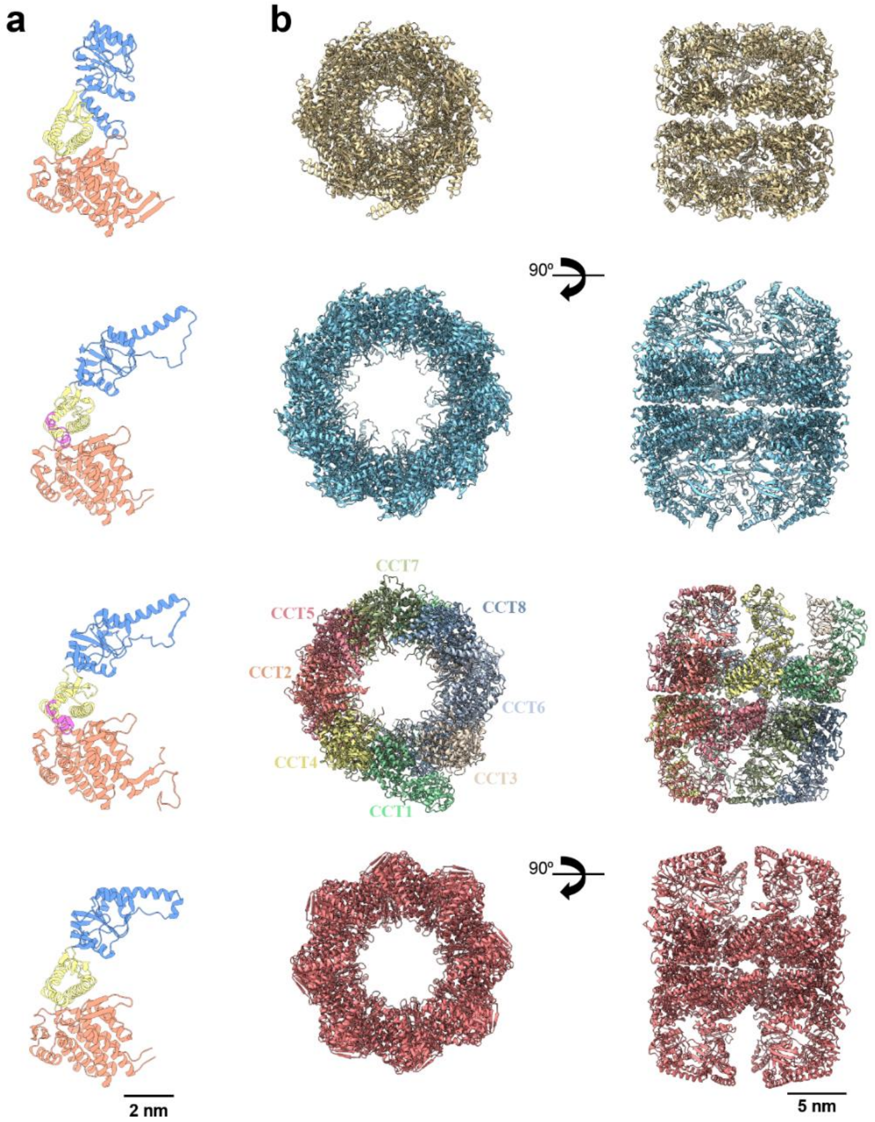


*Supplementary Fig. 1. Atomic structures of the monomer groups I to III chaperonins and orthogonal views of their corresponding assemblage.*

**a** Atomic structure of the monomer of a group I chaperonin (*Hydrogenobacter thermophilus*, PDB: 8WUX), group II thermosome (*Saccharolobus solfataricus*, PDB:4XCD), group II CCT (*Homo sapiens*, PDB: 6QB8) and group III (*Carboxydothermus hydrogenoformans*, PDB: 5X9U). The color code indicates the classical three domains of a chaperonin monomer (orange, equatorial domain; yellow, Intermediate domain; blue, apical domain). The sequence colored in pink corresponds to the sensing loop. **b** Two orthogonal views (left, top view; right; side view) of (from top to bottom) group I GroEL, group II thermosome from *Saccharolobus solfataricus*, group II human CCT and the group III chaperonin from *Carboxydothermus hydrogenoformans*.

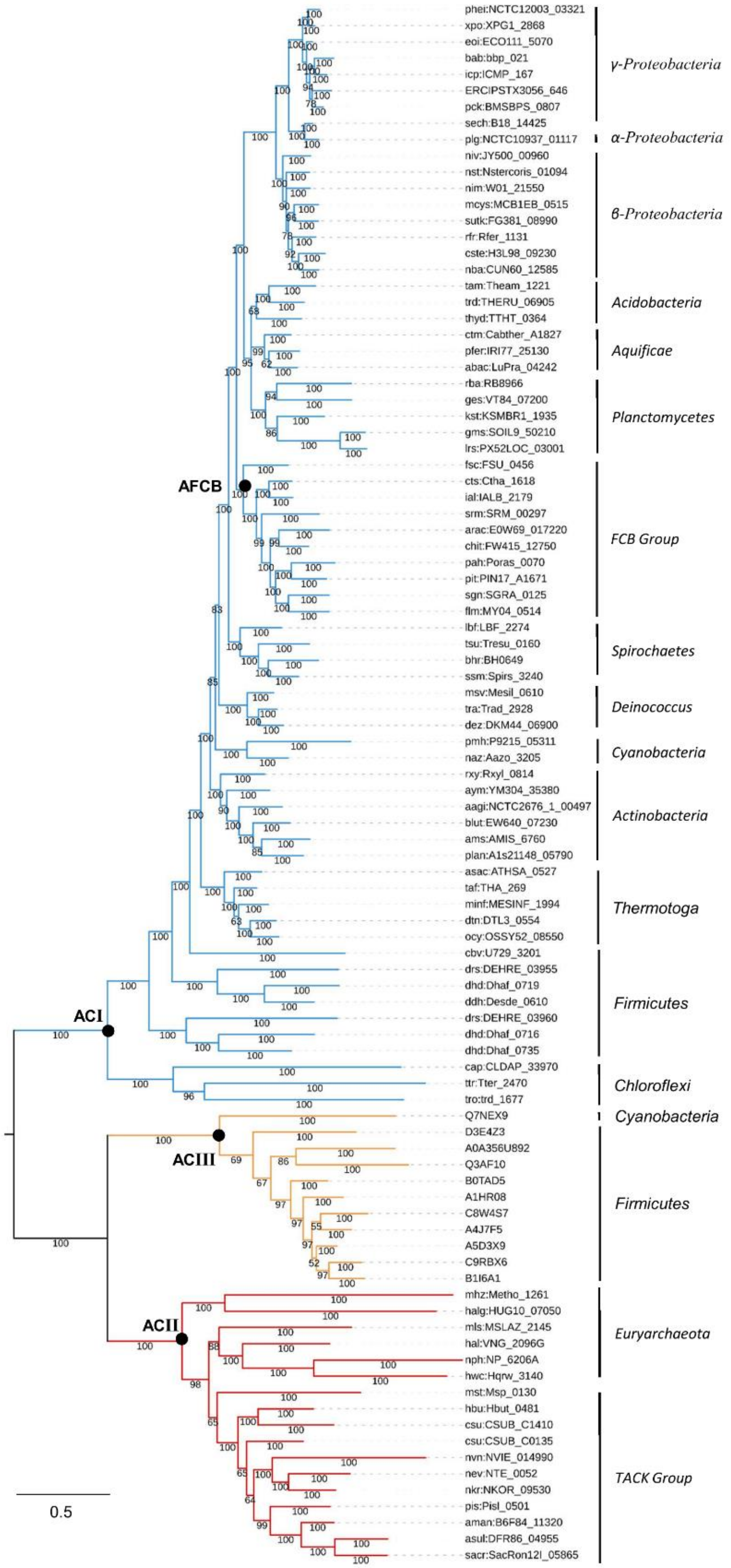
phei:NCTC12003_03321
xpo:XPG1_2868
eoi:ECO111_5070
bab:bbp_021
icp:ICMP_167
ERCIPSTX3056_646
pck:BMSBPS_0807
sech:B18_14425
plg:NCTC10937_01117
niv:JY500_00960
nst:Nstercoris_01094
nim:W01_21550
mcys:MCB1EB_0515
sutk:FG381_08990
rfr:Rfer_1131
cste:H3L98_09230
nba:CUN60_12585
tam:Theam_1221
trd:THERU_06905
thyd:TTHT_0364
ctm:Cabther_A1827
pfer:IRI77_25130
abac:LuPra_04242
rba:RB8966
ges:VT84_07200
kst:KSMBR1_1935
gms:SOIL9_50210
lrs:PX52LOC_03001
fsc:FSU_0456
cts:Ctha_1618
ial:IALB_2179
srm:SRM_00297
arac:E0W69_017220
chit:FW415_12750
pah:Poras_0070
pit:PIN17_A1671
sgn:SGRA_0125
flm:MY04_0514
lbf:LBF_2274
tsu:Tresu_0160
bhr:BH0649
ssm:Spirs_3240
msv:Mesil_0610
tra:Trad_2928
dez:DKM44_06900
pmh:P9215_05311
naz:Aazo_3205
rxy:Rxyl_0814
aym:YM304_35380
aagi:NCTC2676_1_00497
blut:EW640_07230
ams:AMIS_6760
plan:A1s21148_05790
asac:ATHSA_0527
taf:THA_269
minf:MESINF_1994
dtn:DTL3_0554
ocy:OSSY52_08550
cbv:U729_3201
drs:DEHRE_03955
dhd:Dhaf_0719
ddh:Desde_0610
drs:DEHRE_03960
dhd:Dhaf_0716
dhd:Dhaf_0735
cap:CLDAP_33970
ttr:Tter_2470
tro:trd_1677
Q7NEX9
D3E4Z3
A0A356U892
Q3AF10
B0TAD5
A1HR08
C8W4S7
A4J7F5
A5D3X9
C9RBX6
B1I6A1
mhz:Metho_1261
halg:HUG10_07050
mls:MSLAZ_2145
hal:VNG_2096G
nph:NP_6206A
hwc:Hqrw_3140
mst:Msp_0130
hbu:Hbut_0481
csu:CSUB_C1410
csu:CSUB_C0135
nvn:NVIE_014990
nev:NTE_0052
nkr:NKOR_09530
pis:Pisl_0501
aman:B6F84_11320
asul:DFR86_04955
sacr:SacRon12I_05865
γ-Proteobacteria
α-Proteobacteria
β-Proteobacteria
Acidobacteria
Aquificae
Planctomycetes
FCB Group
Spirochaetes
Deinococcus
Cyanobacteria
Actinobacteria
Thermotoga
Firmicutes
Chloroflexi
Cyanobacteria
Firmicutes
Euryarchaeota
TACK Group
AFCB
ACI
ACIII
ACII
0.5

## Supplementary Fig. 2. Phylogenetic reconstruction of prokaryotic chaperonins.

**a** Bayesian phylogeny of prokaryotic chaperonins from Group I (blue), Group II (red), and Group III (yellow), reconstructed with MrBayes 3.2.7. The tree was rooted between Group I and Group II, reflecting the widely accepted placement of LUCA between Bacteria and Archaea. Groups II and III cluster together as suggested previously (Techtmann and Robb 2010; Rebeaud et al. 2021). Four nodes were selected for protein resurrection (black circles), namely the Last Common Ancestors of Group I or, for simplification, Ancestral Chaperonin I (ACI), Group II (ACII), Group III (ACII) and FCB Group (AFCB). Node labels represent posterior probabilities (%). Scale bar: substitutions per site.

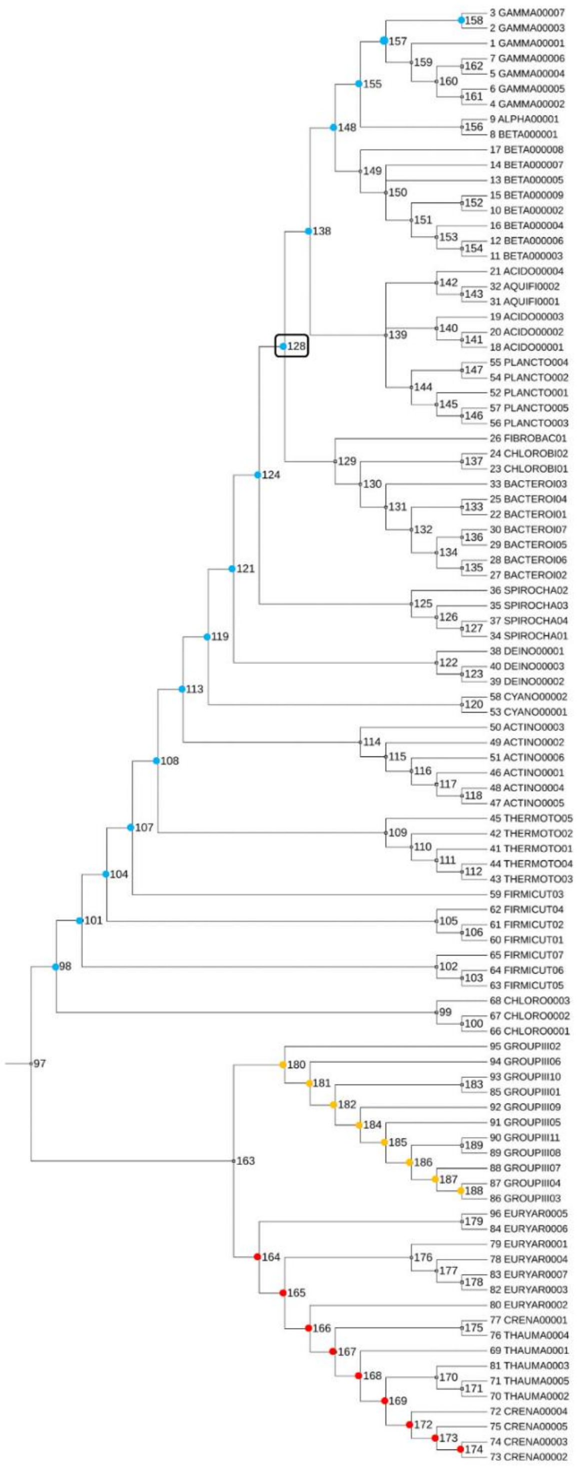


## Supplementary Fig. 3. Cladogram with numbered ancestral nodes

Tree generated by PAML with ancestral nodes numbered (97-189). Colored nodes highlight those used for FoldX-based stability estimations. A black box highlights node 128.

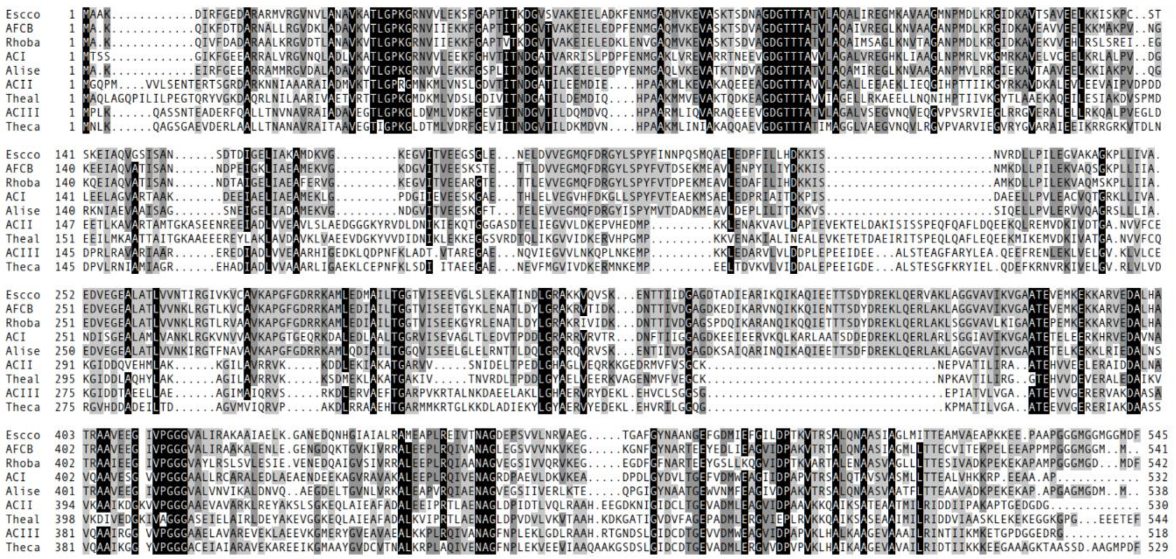

Supplementary Fig. 4: Multiple sequence alignment of best NCBI BLAST matches for resurrected ancestral chaperonin sequences.

The alignment was generated by structural superposition of the corresponding AlphaFold models (https://alphafold.ebi.ac.uk/, AlphaFold Database) using the Dali server (Holm, L. 2022. Dali server: structural unification of protein families. *Nucleic Acids Research* 50(W1), W210-W215) and FoldMason tools (https://github.com/PDB-REDO/foldmason). The resulting alignment was visualized with Belvu (https://sonnhammer.sbc.su.se/Belvu.html) and colored based on sequence identity: >80% (black), 60-80% (grey), and 40-60% (light grey). Sequences included in the analysis are: Chaperonin GroEL from *Escherichia coli* O1:K1 (Escco; UniProt: Q19NJ4_CH602_ECOK1), the last common ancestor of the FCB Group (AFCB), 60 kDa chaperonin from *Rhodothermaceae bacterium* (Rhoba; GenBank: GIV58090.1), the last common ancestor of Group I (ACI), chaperonin GroEL from *Alicyclobacillus sendaiensis* (Alise; GenBank: WP_062305661.1), the last common ancestor of Group II (ACII), thermosome subunit beta from *Thermococcus alcaliphilus* (Theal; GenBank: WP_252742870.1), the last common ancestor of Group III (ACIII), and TCP-1/cpn60 chaperonin from *Thermosinus carboxydivorans* (Theca; GenBank: WP_007289467.1).

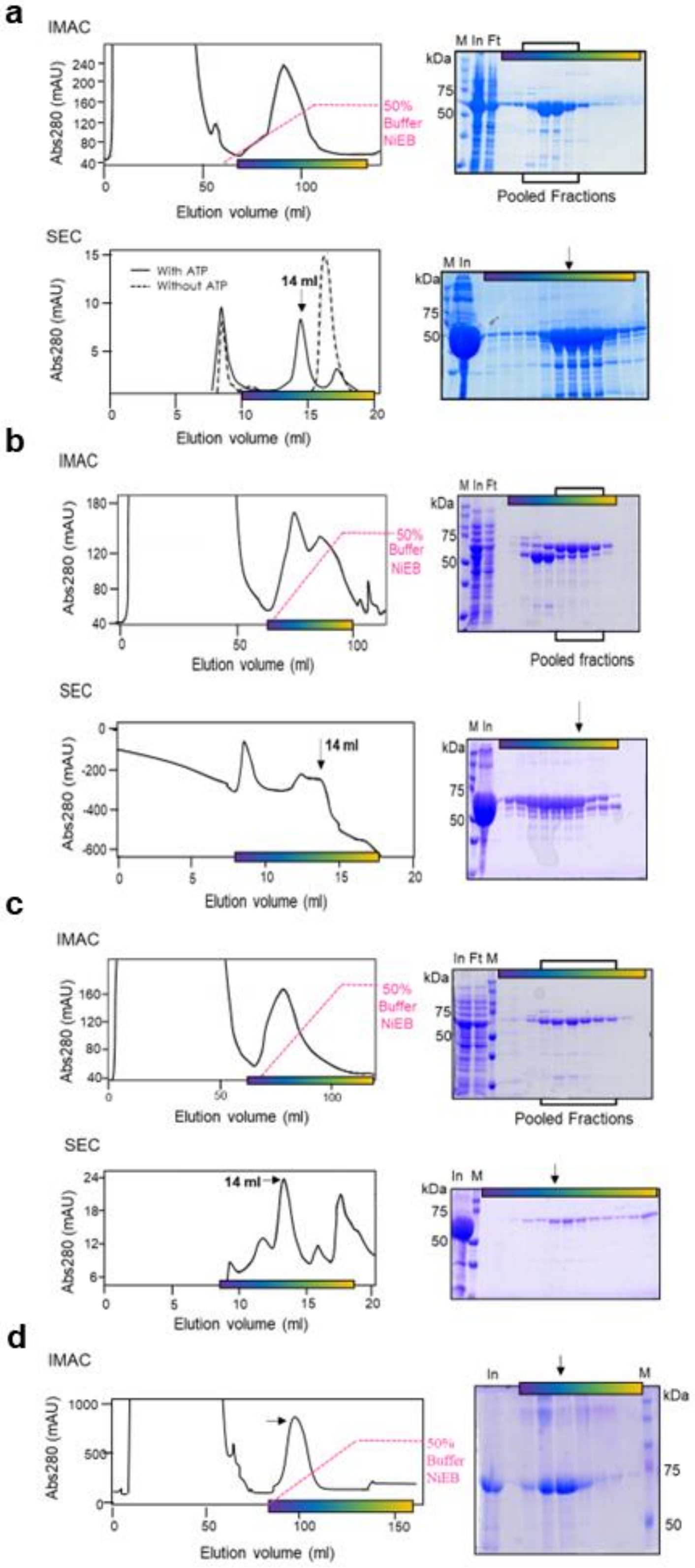
a
IMAC
Abs280 (mAU)
Elution volume (ml)
50% Buffer NiEB
M In Ft
kDa
75
50
Pooled Fractions
SEC
With ATP
Without ATP
14 ml
M In
b
IMAC
Pooled fractions
SEC
14 ml
c
IMAC
In Ft M
Pooled Fractions
SEC
14 ml
In M
d
IMAC
In
M
kDa

## Supplementary Fig. 5. Purification and quality assessment of resurrected ancestral chaperonins

**a-d** Chromatography results and SDS-PAGE analysis are shown for the purification of four ancestral chaperonins ACI, ACII, ACIII, and AFCB, respectively. Proteins were purified from whole cell-lysate by immobilized metal affinity chromatography (IMAC) and size-exclusion chromatography (SEC), using Ni-NTA and Superose® 6 Increase 10/300 GL (GE Healthcare) columns, respectively. After analyzing the fractions, those with the best-assembled and higher amount of chaperonin were pooled, snap frozen in liquid nitrogen, and stored at -20°C. The purest SEC fraction was vitrified and used for 3D CryoEM reconstruction. M – molecular marker; In – input; Ft – flow through.

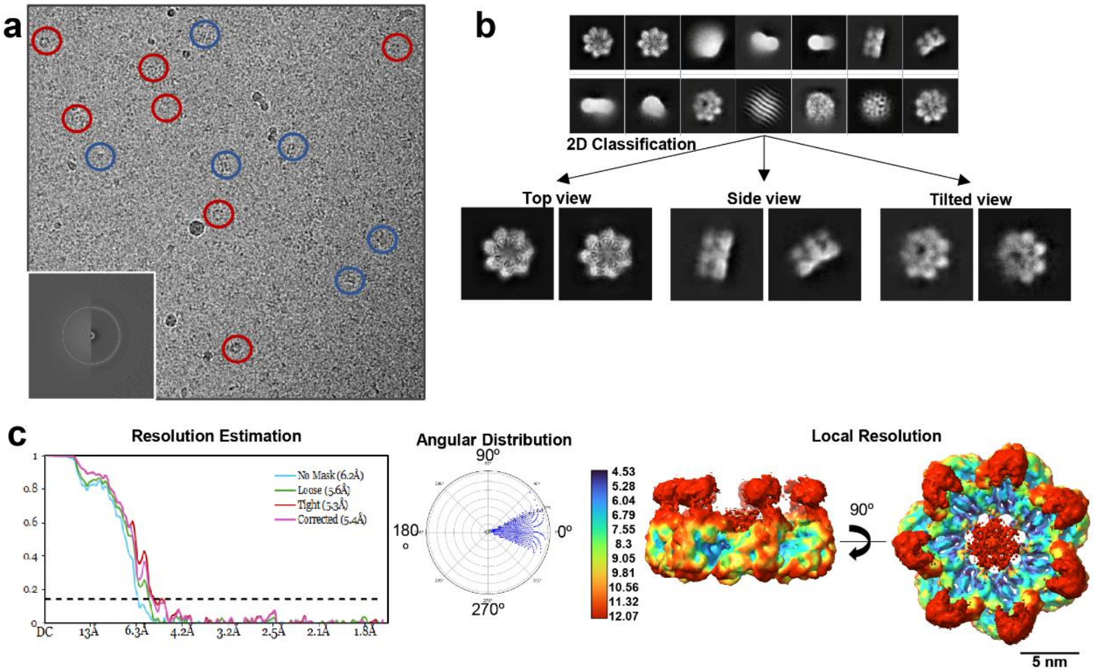


Supplementary Fig. 6 Cryo-EM data processing and workflow of ACI.

**a** Representative cryo-EM micrograph of ACI at ×120,000 magnification. Colored circles highlight different particle orientations: red circles indicate top views, blue circles indicate side views. The inset shows the CTF estimation of the micrograph. **b** 2D classification of cryo-EM particles, revealing different orientations of ACI. Representative top, side, and tilted views are shown. **c** Resolution estimation and angular distribution for ACI 3-D reconstruction. The left panel shows the gold standard Fourier shell correlation (FSC) curve, with the reported resolution indicated as 5.4 Å. The middle panel shows the angular distribution of particles used in the final reconstruction. The right panel displays a local resolution map generated using MonoRes in Xmipp, colored according to local resolution estimates, shown in two orthogonal views.

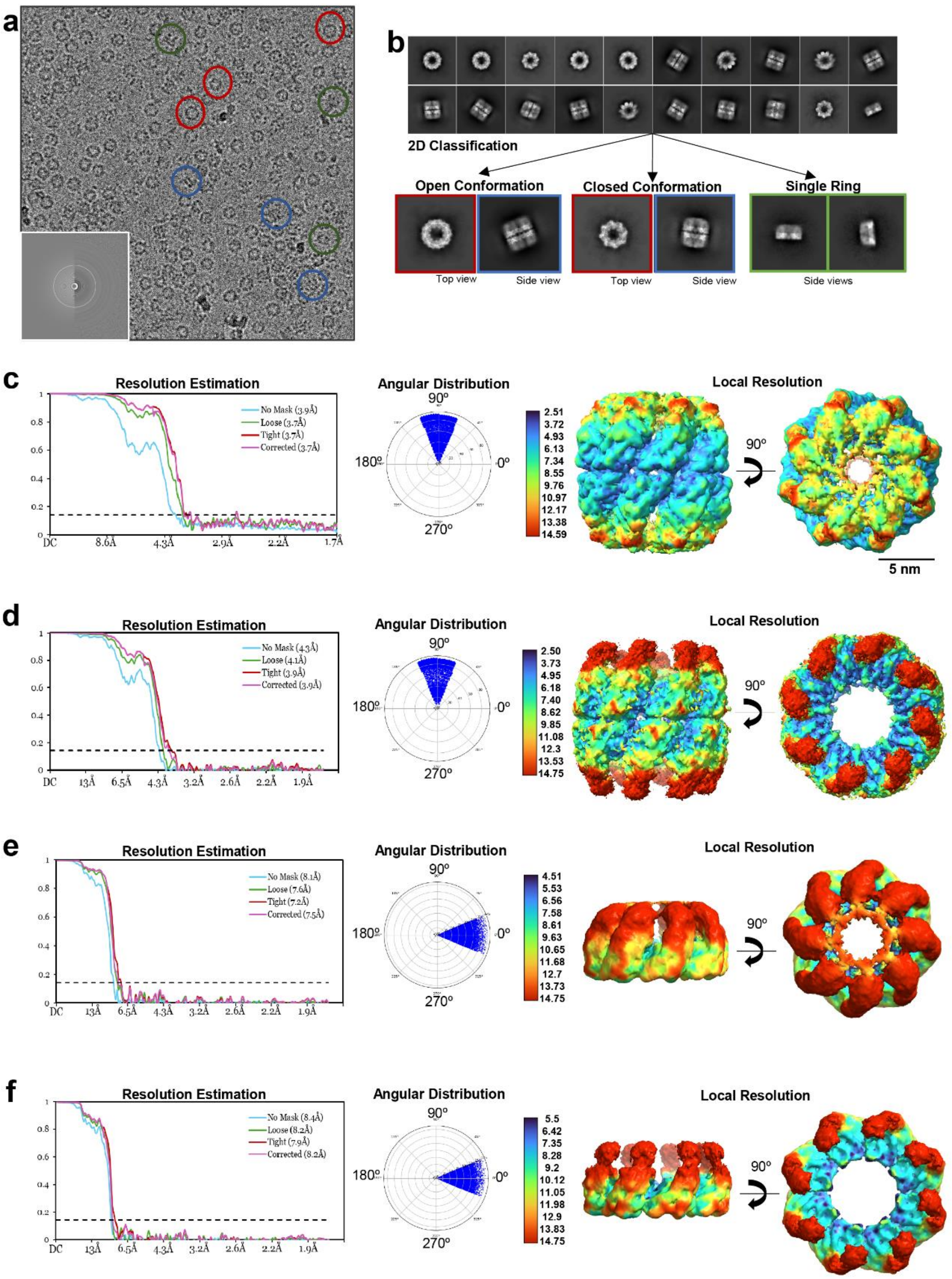


## Supplementary Fig. 7. Cryo-EM data processing and workflow of ACII variants.

**a** Representative cryo-EM micrograph of ACII at ×120,000 magnification. Colored circles highlight different particle orientations: red circles indicate top views, blue circles indicate side views, and green circles indicate single-ring conformations. The inset shows the CTF estimation of the

micrograph. **b** 2D classification of cryo-EM particles, revealing different conformations of ACII, including open and closed conformations, as well as single- and double-ring arrangements. Representative top and side views are shown for each conformation. **c-f** Resolution estimation and angular distribution for double-ring in closed (c) and open (d) conformation and single-ring in closed (e) and open (f) conformation. The left panel shows the gold standard Fourier shell correlation (FSC) curve, with the reported resolution indicated as 3.6 Å (c), 3.9 Å (d), 7.5 Å (e) and 8.2 Å (f). The middle panel shows the angular distribution of particles used in the final reconstruction. The right panel displays a local resolution map generated using MonoRes in Xmipp, colored according to local resolution estimates, shown in two orthogonal views.

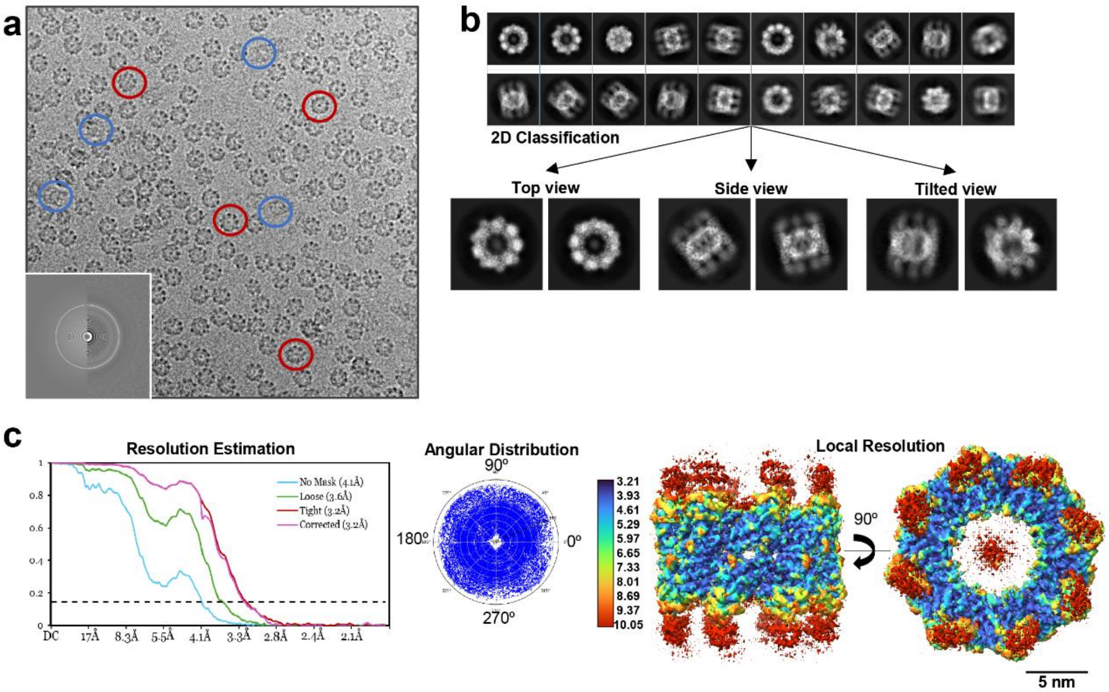


## Supplementary Fig. 8: Cryo-EM data processing and structure validation of ACIII.

**a** Representative cryo-EM micrograph of ACIII at ×130,000 magnification. Colored circles highlight different particle orientations: red circles indicate top views, and blue circles indicate side views. The inset shows the CTF estimation of the micrograph. **b** 2D classification of cryo-EM particles, revealing different orientations of ACIII Representative top, side, and tilted views are shown. **c** Resolution estimation and angular distribution for ACIII 3-D reconstruction. The left panel shows the gold standard Fourier shell correlation (FSC) curve, with the reported resolution indicated as 3.2 Å. The middle panel shows the angular distribution of particles used in the final reconstruction. The right panel displays a local resolution map generated using MonoRes in Xmipp, colored according to local resolution estimates, shown in two orthogonal views.

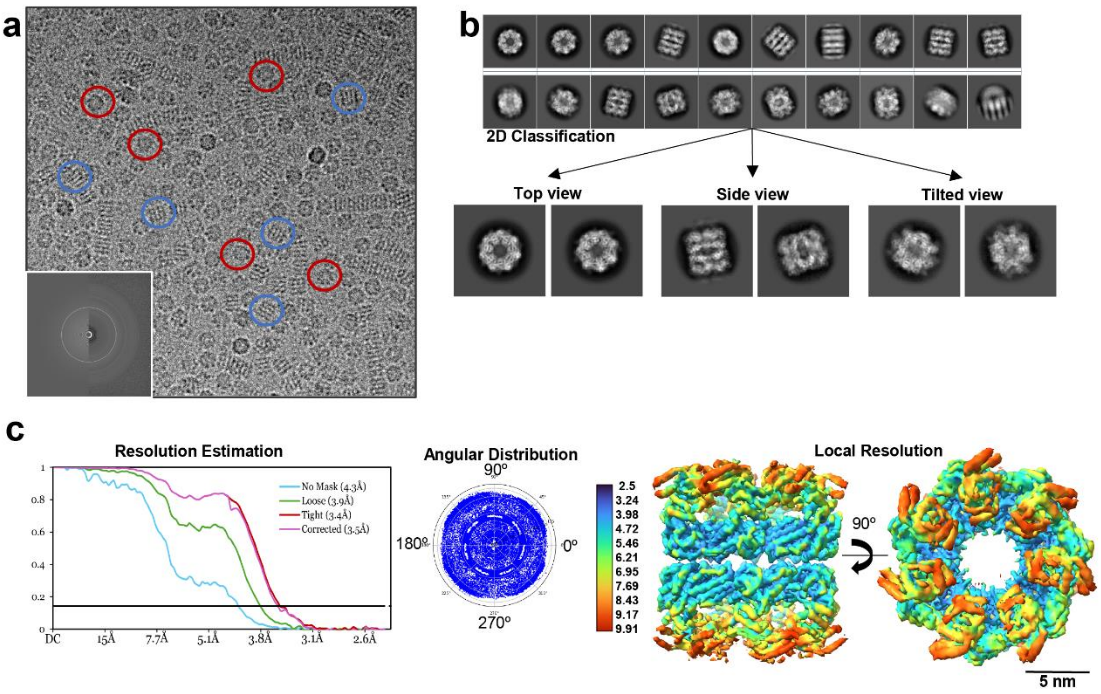


## Supplementary Fig. 9: Cryo-EM data processing and structure validation of AFCB.

**a** Representative cryo-EM micrograph of AFCB at ×120,000 magnification. Colored circles highlight different particle orientations: red circles indicate top views, and blue circles indicate side views. The inset shows the CTF estimation of the micrograph. **b** 2D classification of cryo-EM particles, revealing different orientations of AFCB. Representative top, side, and tilted views are shown. **c** Resolution estimation and angular distribution for AFCB 3-D reconstruction. The left panel shows the gold standard Fourier shell correlation (FSC) curve, with the reported resolution indicated as 3.4 Å. The middle panel shows the angular distribution of particles used in the final reconstruction. The right panel displays a local resolution map generated using MonoRes in Xmipp, colored according to local resolution estimates, shown in two orthogonal views.

## Supplementary Table I. Data acquisition for Resurrected Ancestral Chaperonins

| | ACI | AFCB | ACII | ACIII |
|---|---|---|---|---|
| **Data collection & processing** | | | | |
| Microscope | FEI TALOS Arctica | | | FEI Titan Krios |
| Voltage (KV) | 200 | | | 300 |
| Camera | Falcon 4i | Falcon 3 | Falcon 4i | Gatan K3 |
| Detector mode | Electron-counting | | | |
| Total dose ($e^-/Å^2$) | 40 | 43 | 38 | 48 |
| Frames | 1305 | 40 | 1035 | 40 |
| Dose per frame ($e^-/Å^2$) | 0.038 | 1.075 | 0.029 | 1.2 |
| Defocus range | -0.8 to -2.2 | | | -1 to -2.4 |
| Pixel size (Å) | 0.85 | | | 0.92 |
| Initial number of particles | 117,944 | 245,876 | 2,474,620 | 504,265 |
| Final number of particles | 28,579 | 44,531 | 39,180 | 40,850 |
| Map resolution (Å) | 5.38 | 3.45 | 3.68 | 3.21 |
| FSC threshold | 0.143 | | | |
| Map resolution range (Å) | 4.5-12.1 | 2.5-9.9 | 2.5-9.9 | 3.25-10 |

Supplementary Table II. Posterior probabilities of key residues at the inter-ring interface across ancestral chaperonin nodes

| | **Amino acid (probability)** | | | |
|---|---|---|---|---|
| **Node*** | **R→R** | **D→E** | **A→S** | **E→V** |
| **98** | R (0.89) | D (0.95) | A (0.82) | E (0.48) |
| **101** | R (0.89) | D (0.97) | A (0.72) | E (0.58) |
| **104** | R (0.95) | D (0.97) | A (0.44) | E (0.69) |
| **107** | R (0.96) | D (0.84) | S (0.59) | E (0.69) |
| **108** | R (0.98) | D (0.75) | S (0.79) | V (1) |
| **113** | R (0.99) | E (0.53) | S (0.96) | V (1) |
| **119** | R (0.99) | E (0.69) | S (0.99) | V (1) |
| **121** | R (1) | E (0.94) | S (1) | V (1) |
| **124** | R (1) | E (0.97) | S (1) | V (1) |
| **128** | R (1) | E (1) | S (1) | V (1) |
| **138** | R (1) | E (1) | S (1) | V (1) |
| **148** | R (1) | E (1) | S (1) | V (1) |
| **155** | R (1) | E (1) | S (1) | V (1) |
| **157** | R (1) | E (1) | S (1) | V (1) |

*Node numbers according to Supplementary Fig. 3.

**Supplementary Table III**: Amino acid sequences of the four chaperonin ancestors**.**

| ID | Amino acid sequence |
|---|---|
| **ACI** | MTSSGIKFGEEARRALVRGVNQLADLVKATLGPKGRNVVLEEKFGHVTITNDGATVARRISLPD<br>PFENMGAKLVREVARRTNEEVGDGTTTAVVLAGALVREGHKLIAAGLNPMRLVKGMRKAVELVC<br>EELKRLALPVDGLEELAGVARTAAKDEEIAELIAEAMEKLGPDGIIEVEESKGAETHLELVEGV<br>HFDKGLLSPYFVTEAEKMSAELEDPRIAITDKPISDAEELLPVLEACVQTGRKLLIVANDISGE<br>ALAMLVANKLRGKVNVVAVKAPGTGEQRKDALEDLAALTGGRVISEVAGLTLEDVTPDDLGRAR<br>RVRVTRDNFTIIGGAGDKEEIEERVKQLKARLAATSDDEDREKLQERLARLSGGIAVIKVGAAT<br>ETELEERKHRVEDAVNAVQAAVESGVVPGGGAALLRCARALEDLAEAENDEEKAGVRAVAKALE<br>EPLRQIVENAGRDPAEVLDKVKEADPDLGYDVLTGEFVDMWEAGIIDPAPVTRSALQTAVSVAS<br>MLLTTEALVHKKRPEEAAAP |
| **ACII** | MGQPMVVLSENTERTSGRDARKNNIAAARAIADMVKTTLGPRGMNKMLVNSLGDVTITNDGATI<br>LEEMDIEHPAAKMLKEVAKAQEEEAGDGTTTAVVLAGALLEEAEKLIEQGIHPTTIIKGYRKAV<br>DKALEVLEEVAIPVDPDDEETLKAVARTAMTGKASEENREEIADLVVEAVLSLAEDGGGKYRVD<br>LDNIKIEKQTGGGASDTELIEGVVLDKEPVHEDMPKKLENAKVAVLDAPIEVEKTELDAKISIS<br>SPEQFQAFLDQEEKQLREMVDKIVDTGANVVFCEKGIDDQVEHMLAKKGILAVRRVKKDDLEKI<br>AKATGARVVSNIDELTPEDLGHAGLVEQRKKGEDRMVFVSGCKNEPVATILIRAATEHVVEELE<br>RAIDDALNAVKAAIKDGKVVPGGGAAEVAVARKLREYAKSLSGKEQLAIEAFADALEEIPRTLA<br>ENAGLDPIDTLVQLRAAHEEGDKNIGIDCLTGEVADMLEAGVIDPAAVKKQAIKSATEAATMIL<br>RIDDIIPAKAPTGEDGDG |
| **ACIII** | MPLKQASSNTEADERFQALLTNVNAVRAIADAVEGTLGPKGLDVMLVDKFGEVTITNDGVTILD<br>QMDVQHPAARMLIQVARAQEEEVGDGTTTATVLAGALVSEGVNQVEQGVPVSRVIEGLRRGVER<br>ALELLRKQALPVEGLDDPRLRAVARIAAREREDIADLVVEAARHIGEDKLQDPNFKLADTVTAR<br>EGAENQVIEGVVLNKQPLNKEMPKKLEDARVLVLDDPLEPEEIDEEALSTEAGFARYLEAQEEF<br>RENLEKLVELGVKLVLCEKGIDDTAEELLAEAGIMAIQRVSRKDLERVAEFTGARPVKRTALNK<br>DAEELAKLLGHAERVRYDEKLEHVCLSGGSGEPIATVLVGAATEEVVGERERVAKDAASAVQAA<br>IRGGVVPGGGAAELAVAREVEKLAEEVKGMERYGVEAVAEALKKPLRQIVANAGFNPLEKLGDL<br>RAAHRTGNDSLGIDCDTGEVVDMWEAGVIDPAPVKLHALKAAGEVAAAILRINTIIKMKETGPD<br>GGEDRG |
| **AFCB** | MAKQIKFDTDARNALLRGVDKLADAVKVTLGPKGRNVIIEKKFGAPTITKDGVTVAKEIELEDP<br>FENMGAQMVKEVASKTSDVAGDGTTTATVLAQAIVREGLKNVAAGANPMDLKRGIDKAVEAVVE<br>ELKKMAKPVNGKEEIAQVATISANNDPEIGKLIAEAMEKVGKDGVITVEESKSTETTLDVVEGM<br>QFDRGYLSPYFVTDSEKMEAVLENPYILIYDKKISNMKDLLPILEKVAQSGKPLLIIAEDVEGE<br>ALATLVVNKLRGTLKVCAVKAPGFGDRRKAMLEDIAILTGGTVISEETGYKLENATLDYLGRAK<br>RVTIDKDNTTIVDGAGDKEDIKARVNQIKKQIENTTSDYDREKLQERLAKLAGGVAVIKVGAAT<br>EVEMKEKKARVEDALHATRAAVEEGIVPGGGVALIRAAKALENLEGENGDQKTGVKIVRRALEE<br>PLRQIVANAGLEGSVVVNKVKEGKGNFGYNARTEEYDLIEAGVIDPAKVTRTALQNAASIAGML<br>LTTECVITEKPELEEAPPMPGGGMGGMM |

1 **Supplementary Table IV**. Model refinement and statistics

| MODEL | **ACII Double-Ring in Closed Conformation** | **AFCB Double-Ring in Open Conformation** | **ACIII Double-Ring in Open Conformation** |
|---|---|---|---|
| PDB accession ID | 9RWP | 9RWQ | 9RWR |
| Symmetry imposed | D8 | C1 | C1 |
| **Composition** | | | |
| Chains | 32 | 14 | 16 |
| Atoms | 120944 (Hydrogens: 61056) | 112056 (Hydrogens: 57148) | 81968 (Hydrogens: 41472) |
| Residues | Protein: 7936 Nucleotide: 0 | Protein: 7308 Nucleotide: 0 | Protein: 5456 Nucleotide: 0 |
| Ligands | MG: 16 ADP: 16 | 0 | 0 |
| **Bonds (RSMD)** | | | |
| Length (Å) | 0.006 | 0.006 | 0.006 |
| Angles (º) | 1.239 | 1.338 | 1.309 |
| MolProbity Score | 1.55 | 1.44 | 1.28 |
| Clash score | 6.17 | 4.89 | 5.27 |
| **Ramachandran plot (%)** | | | |
| Outliers | 0.01 | 0 | 0 |
| Allowed | 3.35 | 3.13 | 1.63 |
| Favored | 96.63 | 96.87 | 98.37 |
| Rotamer outliers (%) | 0 | 0.03 | 0.19 |
| Cß outliers (%) | 0 | 0 | 0 |
| **Peptide plane (%)** | | | |
| Cis proline/general | 0.0/0.0 | 0.0/0.0 | 0.0/0.0 |
| Twisted proline/general | 0.0/0.0 | 0.0/0.0 | 0.0/0.0 |

| | | | | | | |
|---|---|---|---|---|---|---|
| CaBLAM outliers (%) | 1.38 | | 2.22 | | 0.68 | |
| **ADP (B-factors)** | | | | | | |
| Iso/Aniso | 59888/0 | | 54908/0 | | 40496/0 | |
| min/max/mean | | | | | | |
| Protein | 67.22/188.38/118.24 | | 43.35/191.62/106.43 | | 39.35/156.94/84.48 | |
| Ligand | 59.79/88.54/79.53 | | --- | | --- | |
| **Occupancy** | | | | | | |
| Mean | 1.0 | | 1.0 | | 1.0 | |
| Occ=1 (%) | 100 | | 100 | | 100 | |
| DATA | | | | | | |
| **Box** | | | | | | |
| Lengths (Å) | 165.62, 165.62, 179.99 | | 162.19, 164.55, 164.55 | | 172.23, 171.31, 116.05 | |
| Angles (Å) | 90.00, 90.00, 90.00 | | 90.00, 90.00, 90.00 | | 90.00, 90.00, 90.00 | |
| Supplied Resolution (Å) | 3.7 | | 3.5 | | 3.2 | |
| Resolution estimates (Å) | Masked | Unmasked | Masked | Unmasked | Masked | Unmasked |
| d FSC (half maps; 0.143) | 3.7 | 3.8 | 3.8 | 3.9 | 3.4 | 3.6 |
| d 99 (full/half/half2) | 2.7/3.0/3.0 | 2.7/2.4/2.4 | 2.8/2.9/2.9 | 2.8/2.7/2.7 | 2.5/2.0/2.0 | 2.5/1.9/1.9 |
| d model | 2.8 | 2.8 | 2.9 | 2.9 | 2.8 | 2.8 |
| D FSC model (0/0.143/0.5) | 1.9/2.7/4.1 | 1.9/2.7/4.1 | 1.8/2.0/3.8 | 1.8/2.0/3.8 | 1.7/2.0/3.6 | 1.7/2.0/3.6 |
| Map min/max/mean | -0.00/2.04/0.02 | | -0.00/2.03/0.04 | | -0.00/1.89/0.03 | |
| **MODEL vs DATA** | | | | | | |
| CC (mask) | 0.69 | | 0.73 | | 0.75 | |
| CC (box) | 0.65 | | 0.72 | | 0.72 | |
| CC (peaks) | 0.65 | | 0.71 | | 0.72 | |
| CC (volume) | 0.69 | | 0.73 | | 0.75 | |
| Mean CC for ligands | 0.78 | | --- | | --- | |
